# *Plasmodium falciparum* Calcium-Dependent Protein Kinase 2 Is Critical for Male Gametocyte Exflagellation but Not Essential for Asexual Proliferation

**DOI:** 10.1128/mBio.01656-17

**Published:** 2017-10-17

**Authors:** Abhisheka Bansal, Alvaro Molina-Cruz, Joseph Brzostowski, Jianbing Mu, Louis H. Miller

**Affiliations:** a Laboratory of Malaria and Vector Research, National Institute of Allergy and Infectious Diseases, National Institutes of Health, Bethesda, Maryland, USA; b LIG Imaging Facility, National Institute of Allergy and Infectious Diseases, National Institutes of Health, Bethesda, Maryland, USA; Washington University School of Medicine

**Keywords:** *Pf*CDPK2, exflagellation, female gametocyte, male gametocyte, mosquito

## Abstract

Drug development efforts have focused mostly on the asexual blood stages of the malaria parasite *Plasmodium falciparum*. Except for primaquine, which has its own limitations, there are no available drugs that target the transmission of the parasite to mosquitoes. Therefore, there is a need to validate new parasite proteins that can be targeted for blocking transmission. *P. falciparum* calcium-dependent protein kinases (*Pf*CDPKs) play critical roles at various stages of the parasite life cycle and, importantly, are absent in the human host. These features mark them as attractive drug targets. In this study, using CRISPR/Cas9 we successfully knocked out *Pf*CDPK2 from blood-stage parasites, which was previously thought to be an indispensable protein. The growth rate of the *Pf*CDPK2 knockout (KO) parasites was similar to that of wild-type parasites, confirming that *Pf*CDPK2 function is not essential for the asexual proliferation of the parasite *in vitro*. The mature male and female gametocytes of *Pf*CDPK2 KO parasites become round after induction. However, they fail to infect female *Anopheles stephensi* mosquitoes due to a defect(s) in male gametocyte exflagellation and possibly in female gametes.

## IMPORTANCE

Despite reductions in the number of deaths it causes, malaria continues to be a leading infectious disease of the developing world. For effective control and elimination of malaria, multiple stages of the parasite need to be targeted. One such stage includes the transmission of the parasite to mosquitoes. Here, we demonstrate the successful knockout of *Pf*CDPK2, which was previously thought to be indispensable for parasite growth in red blood cells. The *Pf*CDPK2 KO parasites are incapable of establishing an infection in mosquitoes. Therefore, our study suggests that targeting *Pf*CDPK2 may be a good strategy to control malaria transmission in countries with high transmission. Moreover, molecular understanding of the signaling pathway of *Pf*CDPK2 may provide additional targets for malaria control.

## INTRODUCTION

**FIG 1:**
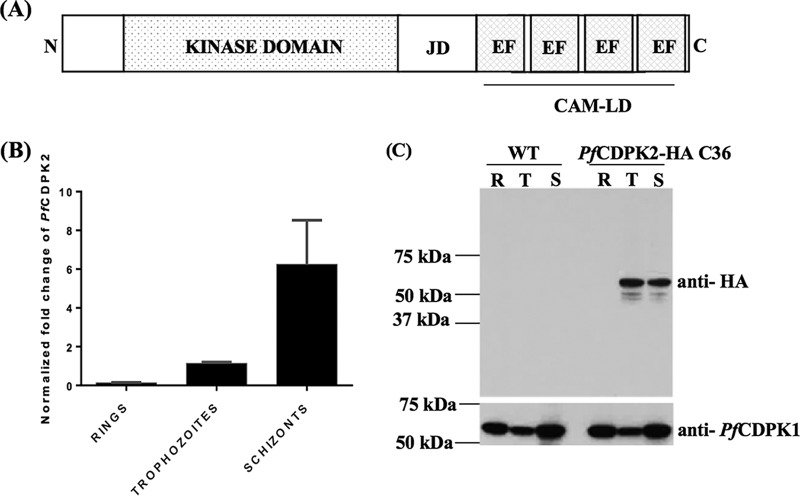
*Pf*CDPK2 is expressed in the asexual stages of *P. falciparum*. (A) Domain organization of *Pf*CDPK2. The figure represents the N-terminal kinase domain, the calmodulin-like domain (CAM-LD), and the junction domain (JD). The CAM-LD contains four EF-hands organized as two EF-hand domain pairs. The domain architecture was constructed using Pfam (http://www.xfam.org). The domains and motifs represented in the cartoon are not to scale. (B) *Pf*CDPK2 transcripts are expressed during the asexual stages of the parasite. The graph shows the normalized transcript expression levels of *Pf*CDPK2 based on real-time quantitative PCR with synchronized rings (9 to 13 hpi), trophozoites (32 to 36 hpi), and schizonts (44 to 48 hpi) of *P. falciparum*. The graph was generated from two independent biological experiments performed in triplicate. Mean values of normalized fold changes are plotted for each stage, and error bars represent standard deviations in the two experiments. (C) The *Pf*CDPK2 protein is expressed in the asexual stage of the parasite. Lysates from synchronized rings (R; 15 to 21 hpi), trophozoites (T; 27 to 33 hpi), and schizonts (S; 42 to 48 hpi) of CDPK2-HA-tagged parasite clone 36 (CDPK2-HA C36) and the WT parasite were separated by SDS-PAGE and probed with anti-HA or anti-*Pf*CDPK1 antibodies. The HA-tagged CDPK2 protein of the expected size (~63 kDa) was detected in the trophozoites and schizonts of CDPK2-HA C36 parasites. WT parasites without the HA tag were used as a negative control. The anti-*Pf*CDPK1 specific antibodies were used to probe a parallel blot as a loading control for the experiment. One representative Western blot is shown from two independent biological replicates. Molecular mass markers are shown.

Calcium is an important second messenger that is involved in multiple signaling cascades at various stages of the *Plasmodium falciparum* life cycle (1). *P. falciparum* calcium-dependent protein kinases (*Pf*CDPKs) act as important mediators in transduction pathways that lead to increased calcium concentrations important for physiological processes. The domain organization of CDPKs typically comprises an N-terminal kinase domain separated from the calmodulin-like domain by a small junction domain (Fig. 1A). Typically, the calmodulin-like domain contains two EF-hand domain pairs at the C terminus. The EF-hand is a helix-loop-helix structure that binds calcium ions in the loop region. The CDPK family in *P. falciparum* includes seven members, of which CDPK1 to -5 conform to the typical domain architecture of the family. *Pf*CDPK1 has been shown to play a role in egress of merozoites from mature schizonts (2, 3), phosphorylation of motor complex proteins (4), and invasion of merozoites into red blood cells (RBCs) (5, 6). *Pf*CDPK4 and *Pf*CDPK5 have been demonstrated to play important roles in male gametocyte exflagellation (7) and egress of merozoites from schizonts (8), respectively.

CDPK2 is an interesting member of the CDPK family in *P. falciparum*, since it does not have a homologue in other human malarial parasites, as evident from information contained within the database PlasmoDB (http://www.plasmodb.org) and a manual BLAST search. CDPK2 homologues are present in the *Plasmodium* species *P. gaboni*, *P. reichenowi*, *P. gallinaceum*, and *P. relictum*. The expected size of the CDPK2 protein is 59 kDa and was shown to exhibit calcium-dependent kinase activity in an *in vitro* phosphorylation assay (9). Failure to disrupt *Pf*CDPK2 using the traditional double homologous recombination strategy suggested an indispensable role of the kinase in the asexual stages of the malaria parasite (10). Treeck et al. (11) identified phosphopeptides for CDPK2 in mature schizont-stage parasites, suggesting that the protein becomes phosphorylated *in vivo*. However, the physiological significance of *Pf*CDPK2 phosphorylation is not known. A previous study predicted the putative substrates of *Pf*CDPK2 based on results of an *in vitro* phosphorylation assay with a nonnatural substrate, myelin basic protein (MBP) (12).

Here, we report successful disruption of the *Pf*CDPK2 gene by using the clustered regularly interspaced short palindromic repeat (CRISPR)–CRISPR-associated protein 9 (CRISPR/Cas9) gene-editing technique. In this study, we show that *Pf*CDPK2 knockout (here referred to as KO) parasites have normal asexual growth similar to that of wild-type (WT) parasites. The KO parasites undergo gametocytogenesis; however, they are defective in male gametocyte exflagellation, a process that results in formation of eight highly motile, flagellated male gametes from a single mature male gametocyte. The first observation of the exflagellation event in blood from malaria patients was made in 1880 by Charles Louis Alphonse Laveran, who realized malaria was caused by a protozoa (13). During exflagellation, the male gametocytes become round, the parasitophorous vacuolar membrane disintegrates, and the RBC membrane ruptures, releasing the male gametes (14, 15). The *in vitro* exflagellation process is completed within 10 to 15 min of induction, during which the parasite undergoes three rounds of nuclear division (16). Importantly, the KO parasites fail to form oocysts in female *Anopheles stephensi* mosquitoes.

## RESULTS

### *Pf*CDPK2 is expressed in the asexual blood stages of the parasite.

Template cDNA prepared from synchronized ring-stage (9 to 13 h postinvasion [hpi]), trophozoite-stage (32 to 36 hpi), and schizont-stage (44 to 48 hpi) wild-type (WT) parasites was used to perform real-time quantitative PCR (RT-qPCR) with the *Pf*CDPK2 (PlasmoDB no. PF3D7_0610600) gene-specific primer pair CDPK2RTF and CDPK2RTR (primer sequences are provided in Table S1 in the supplemental material). When normalized to the expression levels of two *P. falciparum* housekeeping genes, one for glyceraldehyde-3-phosphate dehydrogenase (*Pf*GAPDH) and the other for threonine-tRNA ligase (*Pf*ThrRS), the *Pf*CDPK2 transcript level is extremely low in rings (0.13 ± 0.02; mean ± standard deviation [SD]) and rises at the trophozoite stage (1.14 ± 0.04); it peaks in the schizont stage (6.25 ± 1.61) (Fig. 1B). Samples prepared with no reverse transcriptase (NRT), which were used to rule out the carryover of DNA contamination in the cDNA prepared from the respective stages of the parasites, did not amplify with the *Pf*GAPDH-specific primers (data not shown).

10.1128/mBio.01656-17.8 TABLE S1  Details of primers used in the study. Download TABLE S1, DOCX file, 0.02 MB. Copyright © 2017 Bansal et al. 2017 Bansal et al. This content is distributed under the terms of the Creative Commons Attribution 4.0 International license.

We generated a transgenic parasite with a 3× hemagglutinin (HA) tag at the C terminus of the CDPK2 gene (see Fig. S1) to measure the expression of the CDPK2 protein by Western blotting with different asexual stages of the parasite when we used hemagglutinin-specific (anti-HA) antibodies. CDPK2 protein was detected in trophozoites (27 to 33 hpi) and schizonts (42 to 48 hpi) but not in ring-stage parasites (15 to 21 hpi) (Fig. 1C), consistent with the mRNA expression data. The WT parasites were used as a negative control and did not react with the anti-HA antibodies (Fig. 1C). A parallel Western blot assay was also developed with *Pf*CDPK1-specific antibodies as a loading control for the parasite proteins (Fig. 1C, bottom).

10.1128/mBio.01656-17.2 FIG S1  Generation of HA-tagged CDPK2 parasites. (A) Schematic representation of the strategy used for tagging the endogenous *Pf*CDPK2 gene at the C terminus. The WT locus and the pCAM-BSD plasmid with blasticidin-S deaminase (BSD) selection cassettes are shown. A commercially synthesized *Pf*CDPK2 gene with the start codon (ATG) replaced with TAACG (ATG/TAACG), a homology arm, and the optimized gene sequence are shown. A 3× HA tag was attached at the 3′ end, followed by the 3′ UTR of the *Plasmodium yoelii* chloroquine transporter (*Py*crt 3′ UTR). Selection with BSD integrated the plasmid at the desired site, resulting in a functional copy of an HA-tagged *Pf*CDPK2 gene and a promoterless, nonfunctional CDPK2. (B) Diagnostic PCR for verification of site-specific integration of the plasmid. The positions of the oligonucleotides used for the diagnostic PCR are shown in panel A. A common forward oligonucleotide (F1) was used for all amplification reactions. The reverse oligonucleotides (R8 and R9) are specific for the synthetic gene sequence that results in amplification with F1 only in the transgenic parasite clones (CDPK2-HA C1 and CDPK2-HA C2) and not in the WT. The amplicons with expected sizes of 1,171 and 1,708 bp were obtained with F1-R8 and F1-R9, respectively. The reverse oligonucleotide (R8WT), specific for the WT sequence, was amplified (1,171 bp) only in the WT parasite and not in the CDPK2-HA-tagged parasite clones. Molecular size markers are indicated. Download FIG S1, TIF file, 0.8 MB. Copyright © 2017 Bansal et al. 2017 Bansal et al. This content is distributed under the terms of the Creative Commons Attribution 4.0 International license.

### Disruption of the *Pf*CDPK2 gene.

**FIG 2:**
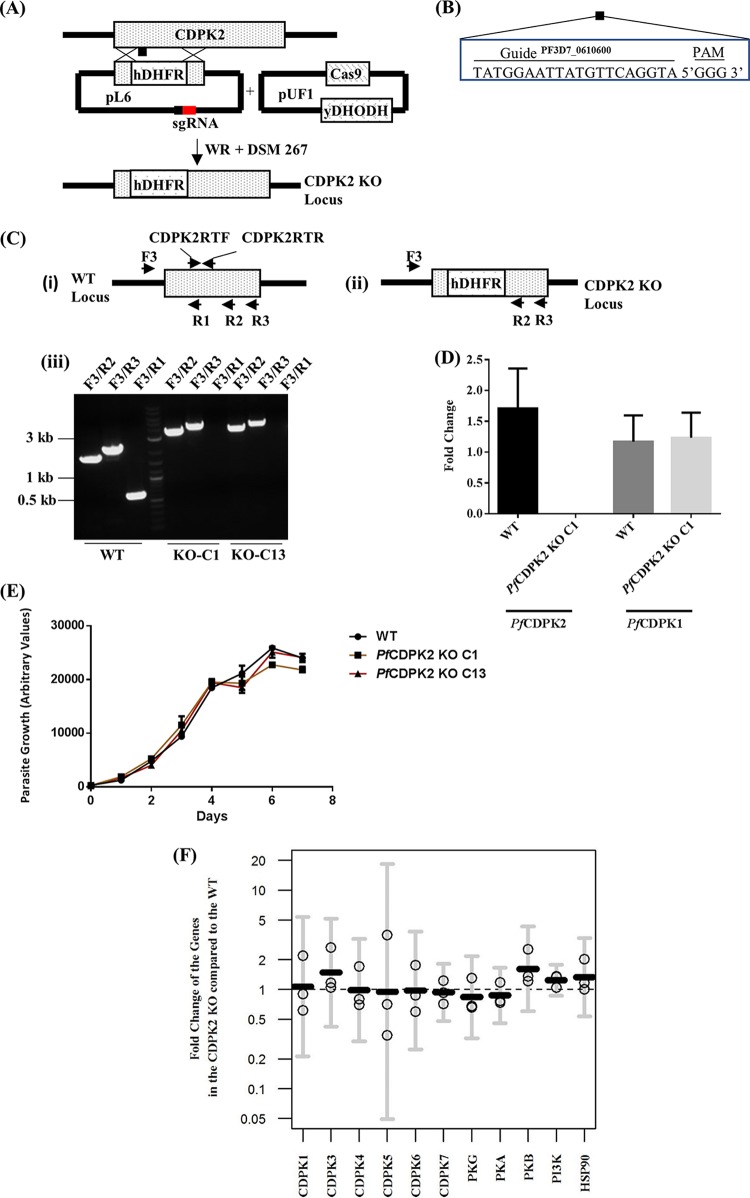
Disruption of the *Pf*CDPK2 gene via CRISPR/Cas9. (A) The diagram represents the strategy for disrupting the *Pf*CDPK2 gene. The single guide RNA (sgRNA) included the guide region specific for targeting the kinase domain of the *Pf*CDPK2 gene cloned in the pL6 plasmid. The Cas9 endonuclease was expressed from the pUF1 plasmid. The pL6 and the pUF1 plasmids with human dihydrofolate reductase (hDHFR) and yeast dihydroorotate dehydrogenase (yDHODH) markers were selected with WR 99210 and DSM 267 drugs, respectively. The CDPK2 KO locus shows the kinase domain replaced with hDHFR. (B) The sequence of the guide region (20 nucleotides) and the PAM. (C) Verification of *Pf*CDPK2 disruption by diagnostic PCR. The positions of the oligonucleotides used for the verification of *Pf*CDPK2 disruption are shown for the WT (i) and CDPK2 KO locus (ii). PCR verification of *Pf*CDPK2 KO clone C1 (KO-C1) and clone C13 (KO-C13) parasite (iii) is shown. A common forward oligonucleotide (F3) specific to the 5′-UTR of *Pf*CDPK2 was used for all of the amplifications. Reverse oligonucleotide R1 specific for the deleted region of the kinase domain amplify only in the WT and not in KO-C1 or KO-C13. Reverse oligonucleotides R2 and R3 yielded amplicons bigger in size in KO-C1 and KO-C13 than in the WT parasite. Molecular size markers are shown. (D) The CDPK2 transcript is not expressed in the KO parasite. The graph shows the mean fold change in transcription for *Pf*CDPK2 and *Pf*CDPK1 in the WT and KO C1 parasites based on real-time quantitative PCR. *Pf*CDPK1 was used as a control for the template cDNA and the experimental conditions. WT was used as a control for the RT-qPCR experiment. The graph was generated from three independent experiments done in triplicate. The error bars represent standard deviations for three experiments. (E) *Pf*CDPK2 KO asexual parasites grew as rapidly as WT parasites. The graph shows similarities in the growth rate profiles of the WT and the two different clones of CDPK2 KO (C1 and C13) over a period of 7 days. The arbitrary fluorescence units, a proxy for parasite growth, on the *y* axis are plotted against days on the *x* axis. The graph shows representative results of three independent biological experiments done in triplicate. The error bars represent standard deviations. (F) RT-qPCR results for expression of 11 genes in CDPK2 KO compared to WT parasites. The expression levels of the 11 different genes (for 10 kinases and HSP90) in the WT and CDPK2 KO C1 parasites were evaluated. The graph shows the fold change of gene expression levels in the CDPK2 KO parasites relative to the WT parasites. The graph was generated using the R statistical analysis package (version 3.3.2) with data from three independent biological experiments done in triplicate. Each circle for a gene represents the arithmetic mean for one of three experiments, and the black horizontal line represents the geometric mean from the three data points. The error bars represent 95% confidence intervals on the geometric mean values. The differences in the transcript levels of the 11 genes in the CDPK2 KO parasites versus those in the WT parasites were not statistically significant.

The endogenous *Pf*CDPK2 gene was disrupted using CRISPR/Cas9 (Fig. 2). The kinase domain was replaced with a human dihydrofolate reductase (hDHFR) cassette by targeting Cas9 endonuclease to make a specific double-stranded break in the kinase domain via a specific 20-nucleotide guide sequence adjacent to the protospacer adjacent motif (PAM; 5′-GGG-3′) (Fig. 2A and B). The KO parasites were cloned by limiting dilution, and the deletion of the kinase domain was verified in the cloned parasites by using a diagnostic PCR with oligonucleotides specific for different regions of the *Pf*CDPK2 gene (Fig. 2C). The reverse oligonucleotide, R1 was selected for the region of the kinase domain deleted in the KO parasites. The oligonucleotide pairs F3 and R2 or F3 and R3 resulted in amplicons of expected sizes of 1,835 or 2,316 bp and 3,420 or 3,901 bp, respectively, for the WT and two clones of CDPK2 KO (KO C1 and KO C13) parasites, respectively (Fig. 2C, panel iii). The oligonucleotide pair F3/R1 resulted in an amplicon of the expected size of 605 bp with the WT but not with the KO C1 and KO C13 parasites (Fig. 2C, panel iii). Disruption of *Pf*CDPK2 was further confirmed by RT-qPCR. For this, the cDNA prepared from schizonts (42 to 48 hpi) of KO C1 and WT parasites was used to perform RT-qPCR with the oligonucleotides CDPK2RTF and CDPK2RTR (sequences are provided in Table S1), which are specific for the deleted kinase domain. The CDPK2RTF/CDPK2RTR primer pair was amplified in the WT but not in the KO C1 parasites (Fig. 2D). *Pf*CDPK1-specific oligonucleotides CDPK1RTF and CDPK1RTR (see Table S1 for sequences) were used as a control for the cDNA preparation (Fig. 2D). NRT for WT and KO C1 did not result in amplification with the *Pf*ThrRS-specific primer pair (data not shown). Thus, the kinase domain of *Pf*CDPK2 is deleted in the KO.

### *Pf*CDPK2 is not essential for asexual proliferation of the parasites *in vitro*.

A SYBR green assay was used to compare the growth rates of two different clones of KO (C1 and C13) with the growth rate of WT parasites. The proliferation of the parasites was followed for 7 days, with sampling every 24 h. The growth rates of the KO parasite clones were similar to that of the WT parasite (Fig. 2E), indicating that *Pf*CDPK2 is not essential for the proliferation of the parasite *in vitro*. Next, we tested whether overexpression of other kinases may compensate for the loss of *Pf*CDPK2 in the KO parasites. For this, we tested the transcript levels of 11 different genes (10 kinases and Hsp90) in the KO C1 and the WT parasites at the late schizont stage (42 to 48 hpi). The 10 kinase genes were selected based on an earlier study (17). The HSP90 gene was selected to evaluate the stress response in the KO parasites. The transcript levels of the 11 genes tested were not significantly changed in the KO parasites compared to the WT parasites (Holm adjusted *P* = 1) (Fig. 2F). NRT for the WT and KO parasites did not amplify with the *Pf*ThrRS-specific primer pair (data not shown). It is possible that other kinases (not tested) may compensate for loss of CDPK2 or that the gene is not required for asexual proliferation of the parasite *in vitro* but may be required *in vivo*.

### *Pf*CDPK2 KO parasites undergo gametocytogenesis.

**FIG 3:**
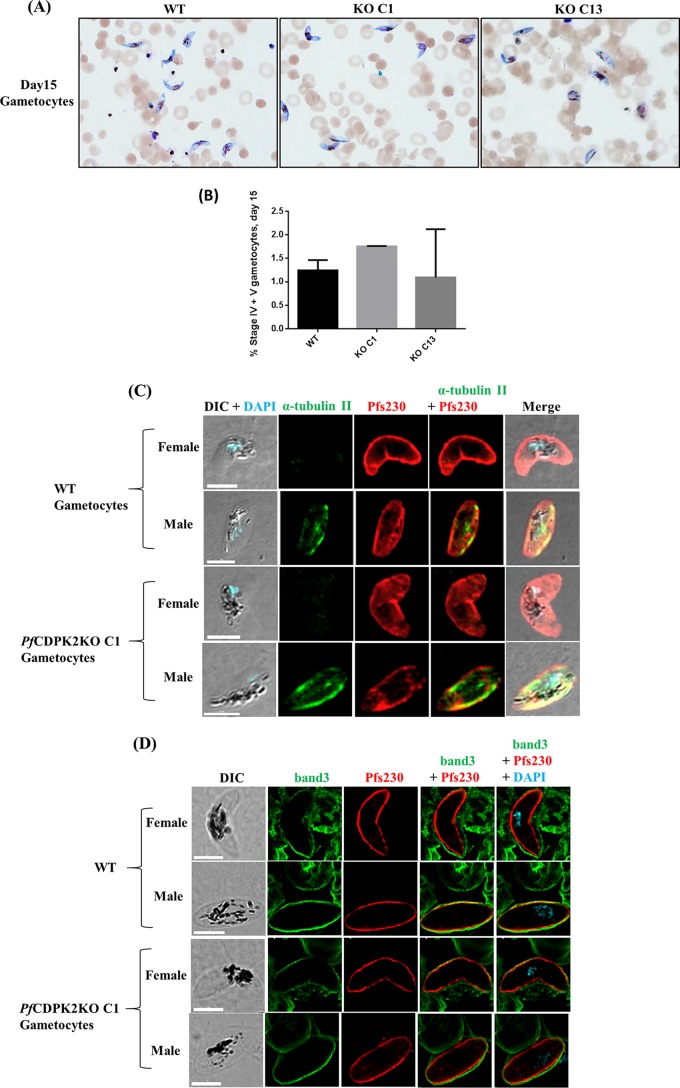
*Pf*CDPK2 KO parasites undergo gametocytogenesis. (A) Two different clones of *Pf*CDPK2 KO parasites, KO C1 and KO C13, along with the WT, were tested for their potential to form gametocytes. Giemsa-stained smears for day 15 gametocytes are shown (see also Table 1). (B) The graph shows the percentages of stage IV and V gametocytes at day 15 for the WT (1.3 ± 0.2), KO C1 (1.8 ± 0.01), and KO C13 (1.1 ± 1) parasites. The graph was generated from two independent experiments performed in triplicate. The error bars represent standard deviations from two experiments. (C) Morphology of the KO gametocytes appeared normal compared to the WT in the IFA. Blood smears of day 15 gametocytes from WT and KO C1 were stained with antibodies against α-tubulin II (green) for male gametocytes and *Pfs*230 (red) for male and female gametocytes. The differential interference contrast (DIC) image was merged with an image showing DAPI staining for nuclear localization. The representative images of the male and female gametocytes of WT and KO C1 are shown. White scale bars, 5 µm. (D) The RBC membrane surrounding the mature gametocytes of KO C1 parasites looks intact by IFA, as in the WT. Mature gametocytes of KO C1 parasites and WT stained with anti-band 3 (green), a surface marker for RBC membrane, and anti-*Pfs*230 (red) antibodies are shown. The mature male and female gametocytes are surrounded by an intact RBC membrane. Representative images of the mature male and female WT and KO C1 gametocytes are shown. White scale bars, 5 µm.

We next evaluated the potential of the KO parasites to undergo gametocytogenesis. For this, KO C1 and KO C13 were induced for gametocytogenesis along with the WT parasites, as described earlier (18, 19). The KO parasites underwent gametocytogenesis and were able to develop into mature gametocytes (Fig. 3; Table 1). The percentage of stage V gametocytes in the KO parasites was similar to that in the WT parasites (Table 1). We also inspected the morphology of the male and female stage V gametocytes in an immunofluorescence assay (IFA) (Fig. 3C and D). The distinction between the male and the female gametocytes was made using previously defined morphological differences in distribution of pigment granules and shape (20) and specific staining of the male gametocytes with anti-α-tubulin II antibodies (21) (Fig. 3C). The mature male and female gametocytes of the KO C1 parasites looked morphologically similar to the WT parasites based on the IFA and the Giemsa smears (Fig. 3A, C, and D). Only the male gametocytes, and not those of females, were stained with the anti-α-tubulin II antibodies (green) (Fig. 3C). The RBC membrane, stained with anti-band protein 3 (green) antibodies, surrounding the stage V male and female gametocytes of the KO C1 parasite appeared intact based on IFA (Fig. 3D).

**TABLE 1:** Exflagellation assay results and mosquito infections with WT and *Pf*CDPK2 KO parasites^a^

Expt no.	Parasite	% stage V gametocytes	Sex ratio (male/female)	No. of fields	No. of exflagellations	% infected mosquitoes (no. infected/total no. dissected)	Median no. of oocysts/midgut (range)
1	WT	1.35	ND	10	87	82.5 (33/40)	4 (0–12)
	*Pf*CDPK2 KO C1	1.52	ND	10	6	0 (0/42)	0 (0)
	*Pf*CDPK2 KO C13	0.84	ND	10	5	0 (0/40)	0 (0)
2	WT	1.30	1:1.9 (70/130)	10	24	82.1 (23/28)	5 (0–24)
	*Pf*CDPK2 KO C1	1.60	1:1.8 (87/160)	45	1	0 (0/37)	0 (0)
3	WT	0.15	1:3 (21/63)	10	17	81.8 (27/33)	3 (0–21)
	*Pf*CDPK2 KO C1	0.96	1:2 (100/211)	10	0	0 (0/31)	0 (0)
4	WT	1.11	1:2.6 (56/144)	16	116	78.9 (15/19)	3 (0–10)
	*Pf*CDPK2 KO C1	0.53	1:2.1 (36/77)	26	0	0 (0/20)	0 (0)
5	WT	1.35	1:2.2 (60/129)	20	295	75 (9/12)	2.5 (0–22)
	*Pf*CDPK2 KO C1	0.78	1:1.7 (85/147)	20	0	0 (0/15)	0 (0)

a Data from the *in vitro* exflagellation assay, a semiquantitative measurement, and mosquito infections for WT and *Pf*CDPK2 KO C1 parasite from five different biological experiments are tabulated here. The exflagellation centers were counted under a bright-field microscope with a 40× objective lens. The 40× fields used for counting the exflagellation centers had what appeared to be a similar number of total RBCs in the WT and *Pf*CDPK2 KO parasites. Importantly, the same cultures of the parasites were used for feeding female *Anopheles stephensi* Nijmegen mosquitoes, and no infection was detected with the *Pf*CDPK2 KO parasites in the five experiments. ND, not determined.

**FIG 4:**
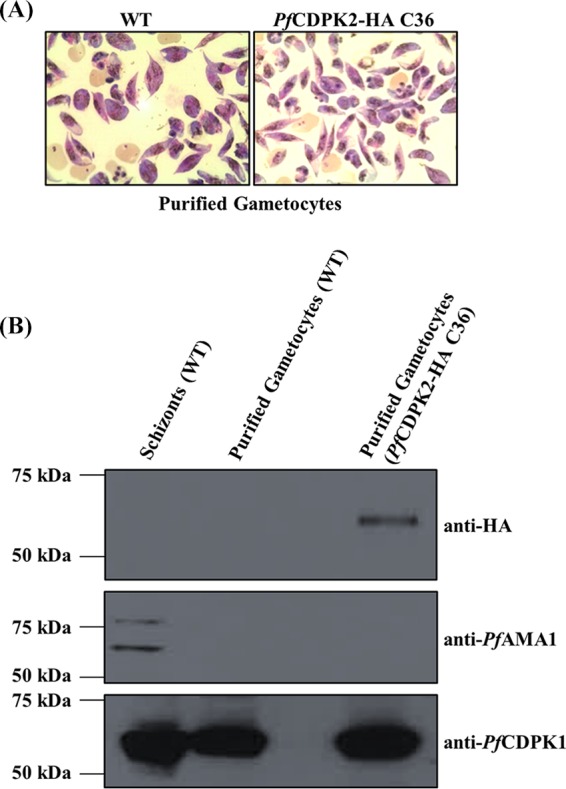
*Pf*CDPK2 protein is expressed in gametocytes. (A) Giemsa smears of purified gametocytes for WTand HA-tagged *Pf*CDPK2 clone 36 (CDPK2-HA-C36) parasites are shown. (B) Lysates prepared from the purified gametocytes of CDPK2-HA-C36 and WT parasites were used for Western blotting. Three parallel blots were probed with anti-HA, anti-*Pf*AMA1, or anti-*Pf*CDPK1 specific antibodies. The anti-HA antibody specifically detected *Pf*CDPK2-HA protein of the expected size (~63 kDa) in the gametocytes from CDPK2-HA-C36 parasites. Lysates of WT schizonts were used as a positive control for anti-AMA1 antibodies. The anti-*Pf*CDPK1 antibodies were used as a loading control. The protein molecular mass markers are shown for each blot. A representative Western blot is shown from two independent experiments.

We used the *Pf*CDPK2-HA-tagged parasites to detect protein expression of *Pf*CDPK2 in the gametocytes. For this, we purified gametocytes and a few gametes from *Pf*CDPK2-HA parasites (Fig. 4A) by killing asexual-stage parasites by using *N*-acetylglucosamine (NAG) (22, 23) and heparin (24), followed by Percoll enrichment, and then we performed a Western blot assay with anti-HA antibodies. The anti-HA antibodies detected *Pf*CDPK2-HA protein of the expected size (~63 kDa) in the purified gametocytes from the *Pf*CDPK2-HA-tagged parasites but not in the WT unlabeled gametocytes (Fig. 4B). A parallel blot was also probed with AMA1-specific (anti-AMA1) monoclonal antibodies to rule out contamination of the gametocyte preparations with asexual-stage parasites. The anti-AMA1 antibodies did not react with the gametocytes (Fig. 4B). The purified schizonts from the WT parasites were used as a positive control for anti-AMA1 antibodies. The antibodies against *Pf*CDPK1 were used as a loading control (Fig. 4B). The results showed that *Pf*CDPK2 is expressed in gametocytes and/or gametes.

### *Pf*CDPK2 is critical for male gametocyte exflagellation and mosquito infection.

**FIG 5:**
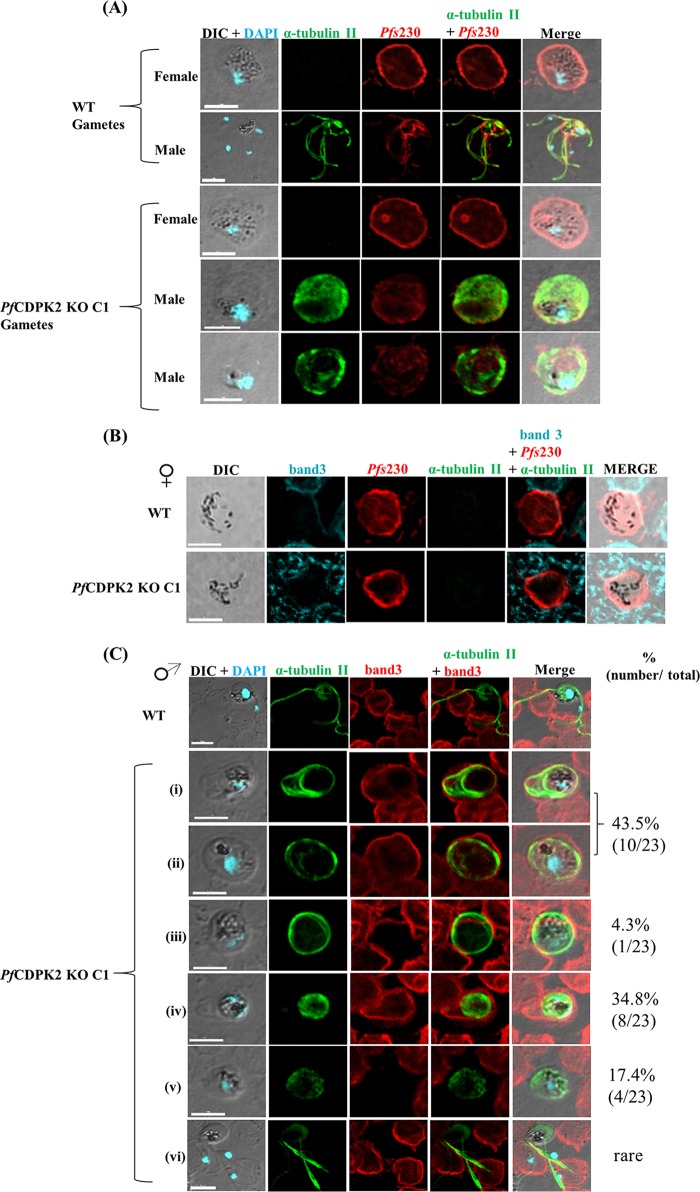
The *Pf*CDPK2 KO parasites are defective in male gametocyte exflagellation (see also Table 1). (A) Blood smears of mature gametocytes induced *in vitro* for 20 min for WT or *Pf*CDPK2 KO C1 (KO C1) parasites were stained for α-tubulin II (green), a male-specific marker, and *Pfs*230 (red), a marker for male and female gametes in an IFA. α-Tubulin II staining showed male gametes emanating from an exflagellating male gametocyte in the WT parasite. The KO parasites were defective for male gametocyte exflagellation. With the KO parasites, two different staining patterns were observed with α-tubulin II (green), one with diffuse staining, indicating a defect in early development of the male gametes, and a second with developed flagellar structures that circled the parasite but did not exflagellate. The female gametes of WT and KO C1 parasites showed similar staining patterns with *Pfs*230 antibodies and looked morphologically similar. (B) The female gametes of KO parasites are able to exit from the RBC. IFA images for WT and KO parasites stained with anti-band 3 (cyan), a marker for RBC membranes, anti-*Pfs*230 (red), and anti-α-tubulin II (green) antibodies are shown. As in the WT parasites, the female gametes of KO C1 do not stain with anti-band 3 (cyan) antibodies. Staining with anti-*Pfs*230 (red) and the absence of staining with the α-tubulin II (green) antibody confirms the identity of the female gametes. (C) Different manifestations of the male exflagellation defect in the KO parasites. The IFA images for WT and KO C1 parasites stained with anti-α-tubulin II (green) and anti-band 3 (red) antibodies are shown. A normal exflagellating male gamete of a WT parasite without band 3 staining is shown. In the *Pf*CDPK2 KO C1 parasites, fully developed flagellar structures stained with α-tubulin II (green) were seen (panels i, ii, and iii) with (panel i and ii [43.5%]) or without (panel iii [4.3%]) band 3 staining. Male gametes of KO C1 parasites with overall and diffuse staining with α-tubulin II (green) are also shown either with (panel iv [34.8%]) or without (panel v [17.4%]) RBC membrane stained with band 3 (red). Rare exflagellating male gametes in the KO C1 parasites appeared thicker and more rigid (panel vi). White scale bars, 5 µm.

**FIG 6:**
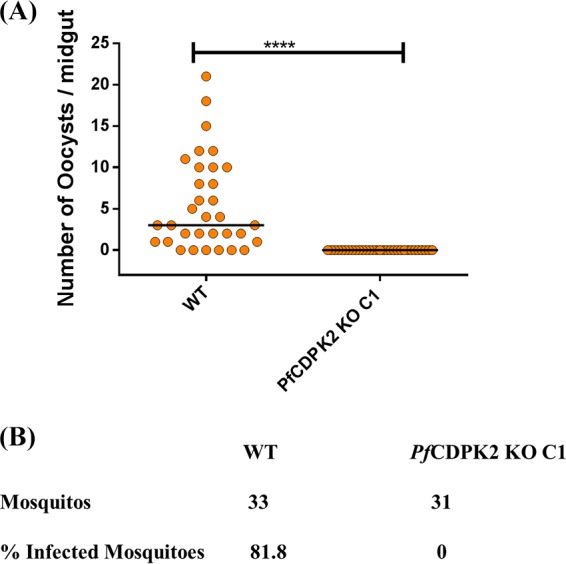
The *Pf*CDPK2 KO parasites were not able to establish infection in mosquitoes. (A) Mosquitoes infected with WT or *Pf*CDPK2 KO C1 (KO C1) parasites were dissected to count the number of oocysts in the midgut. The graph shows the number of oocysts in midguts of mosquitoes infected with WT or KO C1 parasites. The black horizontal lines in the scatter plots denote the median values. A representative graph from five independent experiments is shown. The data (experiment 3 in Table 1) were plotted for 33 and 31 mosquitoes infected with WT and KO C1 parasites, respectively. The Mann-Whitney *t* test was used for calculating statistical significance. ****, *P*  < 0.0001. (B) The percent infected mosquitoes (number of mosquitoes with at least one oocyst per total mosquitoes dissected, × 100) for WT and KO C1 was 81.8% and 0%, respectively.

We tested the ability of the KO parasites to undergo gametogenesis. Day 14 to 16 gametocyte cultures of the WT and the KO C1 parasites were induced for gametogenesis *in vitro* for 20 min by lowering the temperature to room temperature and including human serum in the culture medium. The KO parasites were defective in exflagellation (Fig. 5; Table 1), a process for the exit of male gametes from the male gametocyte. The WT parasite presented 1.7 to 14.7 exflagellations per 40× field in five experiments. In two experiments, we only observed 1 and 6 exflagellation centers in 45 and 10 fields, respectively, in the KO C1 parasites, and none in the subsequent three experiments (Table 1). To further investigate the defect in the KO exflagellation process, we performed an IFA, with anti-α-tubulin II antibodies, on the KO C1 and the WT parasites after 20 min of induction for gametogenesis. The anti-α-tubulin II antibodies specifically recognized the male gametes, but not the female gametes nor the schizont-stage parasites (Fig. S2). In the WT parasites, α-tubulin II antibodies stained the male gametes in the exflagellation center (Fig. 5A and C) that lacked band 3 protein staining (Fig. 5C), confirming exit from RBCs. IFA with the KO C1 parasites showed that the male and female gametes rounded up after induction (Fig. 5). The labeling of the male gametes with the α-tubulin II (green) antibody showed two distinct types of staining patterns: some male gametes showed diffuse staining, while others showed developed flagellar structures (Fig. 5A and C). We also tested the fate of RBC membranes after the induction of the KO C1 parasites by staining for a protein on the RBC surface protein, the band 3 protein (Fig. 5B and C). The female gametes exited from the RBCs normally, as no band 3 (cyan) staining was observed after induction (Fig. 5B). On the other hand, the male gametes showed variation in the disruption of the RBC membrane. Most of the male gametes with developed flagella were not able to exit from the RBCs, as the band 3 (red) staining of the RBC membrane could be detected after induction (Fig. 5C, panels i and ii). However, some male gametes with developed flagella were not surrounded by an RBC membrane (Fig. 5C, panel iii). The same status of the RBC membrane was observed with the male gametes with undeveloped flagella (Fig. 5C, panels iv and v). Very few exflagellations (Fig. 5C, panel vi) or free male gametes (data not shown) were observed in the IFA with the induced KO parasites. However, importantly, when female *Anopheles stephensi* mosquitoes were fed with day 14 to 16 gametocytes of the KO C1 and the WT parasites, no oocysts were observed in the KO-infected mosquitoes in any of the five experiments (Table 1). The WT parasites infected 75 to 82.5% of the mosquitoes (Table 1; Fig. 6A and B). The median oocyst number in *A. stephensi* infected with the WT parasites was 2.5 to 4 (Table 1).

10.1128/mBio.01656-17.3 FIG S2  α-Tubulin II is a male-specific protein. The specificity of the anti-α-tubulin II antibodies (green) was shown in an IFA in the WT parasites, 20 min postinduction. Blood smears with WT parasites were stained with anti-α-tubulin II (green) and *Pfs*230 (red) antibodies. The α-tubulin II (green) antibodies specifically detect male gametes (white arrow) and not the female gamete (black arrow) or mature schizonts (red arrow). The nucleus counterstained with DAPI is shown along with a differential interference contrast (DIC) image. Scale bar, 5 µm. Download FIG S2, TIF file, 0.8 MB. Copyright © 2017 Bansal et al. 2017 Bansal et al. This content is distributed under the terms of the Creative Commons Attribution 4.0 International license.

### CDPK2 is not expressed from the episomal plasmid in *Pf*CDPK2 KO complemented parasites.

We attempted to complement the exflagellation defect in KO parasites via episomal expression of full-length CDPK2. Four plasmid constructs in the pDC2 plasmid background, with CDPK2 gene expression driven by either the CDPK2 5′-untranslated region (5′-UTR; KO^CDPK2 5′-UTR-CDPK2-V5^), *Pfs*230 5′-UTR (KO^*Pf*s230 5′-UTR-CDPK2-V5^) (25), the elongation factor 1α promoter (KO^ef1α CDPK2-V5^) (26), or *Pf*s16 5′-UTR (KO^*Pf*s16 5′-UTR-CDPK2-V5^) (27) were used to transfect the KO parasites (Fig. S3A and S4A). The presence of the episome in the transfected parasites was confirmed by diagnostic PCR after appearance of drug-resistant parasites (Fig. S3B and S4B). Parasites containing an episomal plasmid with the ef1α promoter in the pDC2 plasmid backbone (KO^ef1α-V5^), without the CDPK2 gene, were used as controls (Fig. S3A and S4A). Next, we tested the expression of the *Pf*CDPK2 transcript in the complemented parasites by using cDNA as a template. The CDPK2RTF/CDPK2RTR primer pair amplified the desired 120-bp fragment in the KO^CDPK2 5′-UTR-CDPK2-V5^ and KO^*Pf*s230 5′-UTR-CDPK2-V5^ parasites (Fig. S5). The NRT controls did not result in any amplification, confirming that there was no DNA carryover during cDNA synthesis. It is noteworthy that the transcript of the *Pf*CDPK2 gene was not expressed in the KO^ef1α CDPK2-V5^ parasites (Fig. S5). The sequence of the full open reading frame of *Pf*CDPK2 along with the V5 tag was verified in the plasmids that expressed the transcripts of *Pf*CDPK2 used for complementation (pDC2-CDPK2 5′-UTR-CDPK2-V5, and pDC2-*Pf*s230-5′-UTR-CDPK2-V5).

10.1128/mBio.01656-17.4 FIG S3  A CDPK2-containing episome is present in the CDPK2 KO complemented parasites. (A) Schematic representation of parasites. The WT parasite (i) and the CDPK2 KO parasite (ii) were transfected with pDC2 plasmid containing the CDPK2 gene driven by either the CDPK2 5′-UTR (KO^CDPK2 5′-UTR-CDPK2-V5^) or the *Pf*s230 5′-UTR (KO^*Pf*s230 5′-UTR-CDPK2-V5^) are shown (iii and iv, respectively). CDPK2 KO parasites transfected with pDC2 control plasmid (KO^ef1α-V5^) with the ef1α promoter are shown in panel ii. (B) PCR verification of the episome in the CDPK2 KO parasites. Positions of different oligonucleotides used for confirming the presence of the CDPK2-containing episome in the KO parasites shown in panel A. The oligonucleotide pair Ck2seqHA1/LNHA2 was specifically amplified (amplicon size, 556 bp) in the KO^CDPK2 5′-UTR-CDPK2-V5^ and KO^*Pf*s230 5′-UTR-CDPK2-V5^ parasites. The oligonucleotide pair pDC2A/LNHA2 amplified the desired specific band in the KO^ef1α-V5^ parasites (amplicon size, 286 bp). The oligonucleotide pair F3/R1 (product size, 605 bp) was specifically amplified in the WT and KO^CDPK2 5′-UTR-CDPK2-V5^ parasites. The oligonucleotide pair Ck1F1/Ck1R1wt, used as a positive control, amplified (product size, 628 bp) the region of *Pf*CDPK1 (PlasmoDB no. PF3D7_0217500). A DNA molecular size marker (in kilobases) is shown. Download FIG S3, TIF file, 1 MB. Copyright © 2017 Bansal et al. 2017 Bansal et al. This content is distributed under the terms of the Creative Commons Attribution 4.0 International license.

10.1128/mBio.01656-17.5 FIG S4  A CDPK2-containing episome is present in CDPK2 KO complemented parasites. (A) Schematic representation of parasites. The WT parasite (i) and the CDPK2 KO parasites transfected with pDC2 plasmid containing the CDPK2 gene driven by either ef1α (KO^ef1α-CDPK2-V5^) (ii) or the *Pf*s16 5′-UTR (KO^*Pf*s16 5′-UTR-CDPK2-V5^) (iii) are shown. CDPK2 KO parasites transfected with pDC2 control plasmid with the ef1α promoter (KO^ef1α-V5^) are also shown (iv). (B) PCR verification of the episome in the CDPK2 KO parasites. Positions of different oligonucleotides used for confirming the presence of CDPK2-containing episome in the KO parasites shown in panel A. The oligonucleotide pair Ck2seqHA1/LNHA2 was specifically amplified (amplicon size, 556 bp) in the KO^ef1α-CDPK2-V5^ and KO^*Pf*s16 5′-UTR-CDPK2-V5^ parasites. The oligonucleotide pair pDC2A/LNHA2 amplified in the parasites transfected with the ef1α-containing plasmid (product sizes of 1,831 and 286 bp for KO^ef1α-CDPK2-V5^-containing and KO^ef1α-V5^-containing parasites, respectively). The oligonucleotide pair F3/R1 (product size, 605 bp) was specifically amplified the WT parasite and not in the episome-containing parasites. The oligonucleotide pair GDV1F2/GDV1R was amplified (product size, 2,236 bp) in the full *Pf*GDV1 gene sequence (PlasmoDB no. PF3D7_0935400) in all the parasites showing no deletion of GDV1. The DNA molecular sizes are represented in kilobases. Download FIG S4, TIF file, 1 MB. Copyright © 2017 Bansal et al. 2017 Bansal et al. This content is distributed under the terms of the Creative Commons Attribution 4.0 International license.

10.1128/mBio.01656-17.6 FIG S5  Transcript expression of CDPK2 in the KO parasites complemented with episomal plasmid carrying CDPK2. The cDNA prepared from WT and CDPK2 KO parasites complemented with plasmid carrying the CDPK2 gene with expression driven by either ef1α (KO^ef1α-CDPK2-V5^), CDPK2 5′-UTR (KO^CDPK2 5′-UTR-CDPK2-V5^), or *Pfs*230 5′-UTR (KO^*Pf*s230 5′-UTR-CDPK2-V5^) was used to amplify a 120-bp fragment using primer pair CK2RTF/CK2RTR, which is specific for the deleted region. The transcript of CDPK2 was detected in KO^CDPK2 5′-UTR-CDPK2-V5^ and KO^*Pf*s230 5′-UTR-CDPK2-V5^ but not in the KO^ef1α-CDPK2-V5^ parasites. The NRT controls for each of the cDNA preparations did not show any amplification. DNA molecular size markers (in base pairs) are shown. Download FIG S5, TIF file, 0.8 MB. Copyright © 2017 Bansal et al. 2017 Bansal et al. This content is distributed under the terms of the Creative Commons Attribution 4.0 International license.

The CDPK2 protein was not detected in the late-stage trophozoites (36 to 40 hpi) after transfection in KO^CDPK2 5′-UTR-CDPK2-V5^, KO^*Pf*s230 5′-UTR-CDPK2-V5^, KO^ef1α CDPK2-V5^, and KO^*Pf*s16 5′-UTR-CDPK2-V5^ parasites by Western blotting with anti-V5 tag antibodies (Fig. S6A and B). Parasites with a different genetic background that expressed *Pf*CDPK1-V5 under control of the ef1α promoter from the same plasmid (pDC2) backbone were used as positive controls. Moreover, the V5-tagged CDPK2 protein was not detected in the mature gametocytes of KO^CDPK2 5′-UTR-CDPK2-V5^ or KO^*Pf*s230 5′-UTR-CDPK2-V5^ parasites before or after 20 min of induction for gametogenesis (Fig. S6C). We did not observe any exflagellation in the day 14 gametocytes of KO^CDPK2 5′-UTR-CDPK2-V5^ or KO^*Pf*s230 5′-UTR-CDPK2-V5^ parasites after 20 min of induction, possibly due to the absence of CDPK2 protein expression (data not shown). Moreover, we did not observe any infection in the mosquitoes fed KO^CDPK2 5′-UTR-CDPK2-V5^ or KO^*Pf*s230 5′-UTR-CDPK2-V5^ parasites (data not shown).

10.1128/mBio.01656-17.7 FIG S6  *Pf*CDPK2 protein is not expressed in the *Pf*CDPK2 KO parasites carrying episomes containing the *Pf*CDPK2 gene in either the asexual or sexual stages. (A) Western blot analysis was done with *Pf*CDPK2 KO parasites containing an episome with *Pf*CDPK2-V5 gene expression driven by either CDPK2 5′-UTR (KO^CDPK2 5′-UTR-CDPK2-V5^) or *Pfs*230 5′-UTR (KO^*Pf*s230 5′-UTR-CDPK2-V5^) by using anti-V5 antibodies. (B) A separate Western blot for *Pf*CDPK2-V5 detection with anti-V5 antibodies in KO^ef1α-CDPK2-V5^ and KO^*Pfs*16 5′-UTR-CDPK2-V5^ parasites. WT, CDPK2 KO (KO), and CDPK2 KO containing empty plasmid without *Pf*CDPK2 (KO^ef1α-V5^) were used as negative controls. All the parasites used for the Western blotting were collected 36 to 42 h postinvasion. A parallel blot was probed with anti-*Pf*CDPK1 antibodies. (C) *Pf*CDPK2-V5 expression was tested in the KO^CDPK2 5′-UTR-CDPK2-V5^ and KO^*Pf*s230 5′-UTR-CDPK2-V5^ gametocytes before (BI) and after (AI) 20 min of induction for gametogenesis via anti-V5 antibodies. WT parasites were used as negative controls. Parasites with a different genetic background expressing *Pf*CDPK1-V5 under the ef1α promoter from the same plasmid backbone were used as positive controls. Download FIG S6, TIF file, 0.7 MB. Copyright © 2017 Bansal et al. 2017 Bansal et al. This content is distributed under the terms of the Creative Commons Attribution 4.0 International license.

## DISCUSSION

For continuation of the *Plasmodium* life cycle, the sexually differentiated cells, gametocytes, must be taken up by a mosquito in its blood meal. Within 10 min of uptake, the mature male and female gametocytes differentiate into male and female gametes through a process known as gametogenesis. The gametogenesis process in *P. falciparum* can be induced *in vitro* by lowering the temperature of the external milieu bathing the gametocytes and increasing the pH (28–31). Gametogenesis is a complex and rapid process that requires precise orchestration of various signaling cascades.

In the present study, we showed that *Pf*CDPK2 is not required for *in vitro* asexual proliferation and gametocytogenesis of the malaria parasite *P. falciparum*. However, disruption of *Pf*CDPK2 (KO) caused a defect in exflagellation of the male gametocytes. Previous studies have shown that *Pf*CDPK4 also plays a role in the exflagellation process, as demonstrated using a chemical genetics approach (7, 32). However, the precise role of CDPK4 in *P. falciparum* during exflagellation is not known. A recent report showed that *P. berghei* CDPK4 is involved at multiple stages of genome replication during the exflagellation of male gametocytes (33). *Pf*CDPK4 may have a similar function as its homologue in *P. berghei*. Our results indicate that *Pf*CDPK2 plays a role in the development of the male gametes inside RBCs. The male gametocytes of the KO become rounded after induction. However, the status of the RBC membrane varies. The fact that both undeveloped and developed flagellar structures were visible with anti-α-tubulin II antibodies suggests that *Pf*CDPK2 may have a role in the development of the male gametes. Moreover, *Pf*CDPK2 may also have a role in the exit of male gametes from RBCs, as some gametes with developed flagella had an intact RBC membrane rather than being free of the RBC membrane. *Plasmodium* perforin-like protein 2 (PPLP2) has been shown to play a critical role in the lysis of RBC membranes during exit of gametocytes (34). *Pf*CDPK2 may have a role in PPLP2 activation for exit of male gametocyte during exflagellation. Taken together, our study indicates multiple defects in the male gametocyte exflagellation of the CDPK2 KO parasites, such as RBC membrane lysis and development of the flagella. The fact that very few exflagellation centers are observed in *Pf*CDPK2 KO parasites, along with few free male gametes, suggests that male gametes may be abnormal in the KO parasites. Interestingly the KO parasites were not able to infect female *Anopheles stephensi* mosquitoes in the five independent experiments performed. Taken together, these results indicate that *Pf*CDPK2 may also have a role in the fertilization of the female gametes by male gametes, development of ookinetes, or result in the number of free male gametes being insufficient to establish infection in mosquitoes. The female gametocytes in the KO rounding up process were released from the RBC membrane after induction and looked morphologically similar to the WT parasites in the immunofluorescence assay. We have not tested the functional viability of the female gametes in the KO parasites by using complementation with a parasite line that only produces male gametes as, to our knowledge, none exists. Therefore, it is possible that the female gametes are also not normal and cannot be fertilized.

We attempted to complement the KO parasites by episomal expression of full-length CDPK2. However, we could not express CDPK2 in the KO parasites in the asexual and sexual stages. Depending on the promoter sequence, the expression of CDPK2 from the episomal plasmid was regulated at transcription (KO^ef1α CDPK2-V5^) or posttranscription (KO^CDPK2 5′-UTR-CDPK2-V5^ and KO^*Pf*s230 5′-UTR-CDPK2-V5^), both of which showed message but with no protein detected. The malaria parasite suppresses the transcription of genes by epigenetic silencing of promoter elements (reviewed in references 35, 36, 37). Silencing of the transgene from episomal DNA vectors with an accompanying increase in heterochromatin-associated histone modifications was demonstrated by Riu et al. in mammalian cells (38). It is not known if a mechanism of transgene silencing from episomal DNA exists in *Plasmodium*. Malaria parasites also regulate the expression of genes by translational repression until they are required at a later stage of the parasite life cycle (39–42). The fact that *Pf*CDPK2 transcript was expressed in the KO^CDPK2 5′-CDPK2-V5^ and KO^*Pf*s230 5′-UTR-CDPK2-V5^ parasites without being translated suggests translational repression as a possibility. The sequence of the complete open reading frame of CDPK2 along with the V5 tag was verified, ruling out the possibility of point or frameshift mutations leading to disruption of the V5 tag. It could be that deletion of the CDPK2 gene leads to changes in expression of other genes in the KO parasites and the altered genetic background is not conducive for CDPK2 protein expression. The precise molecular mechanism for the exclusion of CDPK2 protein expression in the KO parasites needs further investigation. The pDC2 plasmid DNA, with the ef1α promoter, that was employed for complementation of the KO parasite was successfully used to express *Pf*CDPK1 in a parasite with a different genetic background. Therefore, it is unlikely that the vector is the problem.

Previous studies with *Pf*CDPK4 (with a small serine gatekeeper residue) showed that bumped kinase inhibitors, such as 1294 and BKI-1, block exflagellation of mature NF54 gametocytes (7, 32). Preincubation of the gametocytes with 1294 or BKI-1 effectively blocked oocyst formation in the mosquito midgut (7, 32). To show that the effect of 1294 on oocyst development was due to *Pf*CDPK4, a transgenic parasite that exogenously expressed a mutant *Pf*CDPK4 (not inhibited by 1294 or BKI-1) with a bulky methionine gatekeeper residue was generated (32). The exflagellation 50% effective concentration shifted from 0.023 µM (for the WT parasites) to 0.292 µM (for the mutant parasites). Sterile protection with 1294 in the WT parasites was achieved at a concentration of 3 µM. However, as the authors of that study also discussed, the higher concentration of 1294 could inhibit other kinases such as *Pf*CDPK1 (32). Since *Pf*CDPK2 has a bulky methionine gatekeeper residue, inhibition by 1294 or BKI-1 is unlikely. Nonetheless, the study showed that it is feasible to develop transmission-blocking drugs with prolonged half-lives in human blood that could potentially be combined with an existing regimen of drugs targeting the asexual stage of the parasite, such as artemisinin combination therapy (ACT). This strategy may be quite useful in countries with high malaria transmission. The present study highlights an important role of *Pf*CDPK2 in the transmission of the malaria parasite from humans to mosquitoes and uncovers transmission to mosquitoes as an additional target in the arsenal of transmission-blocking drugs. It will be interesting to decipher the signaling cascade of CDPK2 and understand how it fits with CDPK4 in overall regulation of the parasite’s male gametocyte exflagellation. This study provides an initial link for CDPK2 with the exflagellation process that may be explored further to better understand the process, as this may provide additional targets to block malaria transmission.

## MATERIALS AND METHODS

### Construction of a plasmid for *Pf*CDPK2 knockout.

The 5′ and 3′ homology arms of lengths 432 and 535 bp, respectively, were amplified using the oligonucleotide pairs Ck2crispr5fwd (5′-ATGCGC***ACTAGT***GAGCTCTAACGGGAAATCAC-3′)/Ck2crispr5rev (5′-ATGCGC***CTTAAG***ATTAAATATATATAATTATCATTTTCATATGTTTCATATAATTTTA-3′) and Ck2crispr3fwd (5′-ATGCGC***GAATTC***ATAACACAAATGACTAAAAGCCATG-3′)/Ck2crispr3rev (5′-ATGCGC***CCATGG***GCAAAATATATAGAGAATAAAGCAGAATG-3′). The bold, italicized sequences in the oligonucleotides are the restriction sites for SpeI, AflII, EcoRI, and NcoI, respectively. The 5′ and 3′ homology arms were cloned in the pL6 plasmid (43) flanking the hDHFR cassette, giving rise to the pL6CK2HR plasmid. The guide sequence of 20 nucleotides (5′-TATGGAATTATGTTCAGGTA-3′) was cloned in the pL6CK2HR plasmid, giving rise to the pL6CK2KO plasmid. The guide sequence was selected by manual curation beside the 5′ homology arm with a PAM sequence, 5′-GG-3′.

### *Plasmodium falciparum in vitro* culture and transfections.

The pL6CK2KO plasmid along with the pUF1 plasmid (43) were used for transfecting the *P. falciparum* NF54 parasites. Fifty micrograms of each plasmid was used for the electroporation of ring-stage parasites, under previously published conditions (44). Briefly, the parasites were synchronized using sorbitol treatment, and the ring-stage parasites at 5% parasitemia were electroporated at 310 V and 950 µF by using a Bio-Rad Gene Pulser II (Bio-Rad Laboratories, Hercules, CA). The transfected parasites were treated with WR99210 (Jacobus Pharmaceutical Company) and DSM 267 (a gift from Margaret A. Phillips and Pradipsinh K. Rathod [45]), at final concentrations of 2 nM and 150 nM, respectively, around 24 h after transfection. The recombinant parasites, obtained from three independent transfections that were pooled and cultured together, were confirmed by a diagnostic PCR with gene-specific oligonucleotides. A forward oligonucleotide (F3) specific for the 5′-UTR region of *Pf*CDPK2 was designed along with three different reverse oligonucleotides (R1, R2, and R3). The relative position of the oligonucleotides is shown in Fig. 2C (panels i and ii). The nucleotide sequences of the oligonucleotides are provided in Table S1.

The *P. falciparum* NF54 parasites were cultured in O^+^ human red blood cells (Virginia Blood Services, Richmond, VA) at 2% hematocrit in RPMI 1640 medium with l-glutamine, 25 mM HEPES, and 50 µg/ml hypoxanthine (KD Medical, Columbia, MD) supplemented with 10% heat-inactivated, O^+^ human serum (Interstate Blood Bank Inc., Memphis, TN), 28 ml of 7.5% sodium bicarbonate (KD Medical, Columbia, MD), and 10 µg/ml gentamycin (Gibco, Thermo, Fisher Scientific, Grand Island, NY) as described earlier (46). For the growth rate measurement experiments, the human serum was replaced with 0.5% Albumax I (Thermo, Fisher Scientific, Grand Island, NY). The parasites were grown in an environment consisting of 5% O_2_, 5% CO_2_, and N_2_ at 37°C.

### RT-qPCR.

The transcript expression levels of 11 different genes (10 kinases and HSP90) were tested by RT-qPCR in the *Pf*CDPK2 KO C1 and WT parasites in the schizont stage (42 to 48 h postinvasion). The sequences of the oligonucleotides used for the RT-qPCR are provided in Table S1. The oligonucleotides were designed to produce short amplicons of 115 to 123 bp with similar melting temperatures. The kinase genes selected were either members of the CDPK family or reported to be in the calcium signaling cascade, or are activated by a second messenger other than calcium ions. RNA was isolated from the synchronous parasites at the schizont stage by using the RNeasy minikit (Qiagen, Valencia, CA). Any contamination of DNA in the purified RNA samples was removed by treatment with the Turbo DNA-free kit (Thermo Fisher Scientific, Grand Island, NY), following the manufacturer’s protocol. The cDNA was prepared from the RNA using SuperScript III first-strand synthesis supermix (Thermo Fisher Scientific, Grand Island, NY) following the manufacturer’s protocol. The RT-qPCR experiment was set up in a 96-well plate to amplify the target gene sequences with the template cDNA, using the iQ SYBR green supermix (Bio-Rad Laboratories, Hercules, CA). The reactions were run on the Bio-Rad CFX Connect instrument with the following program: initial denaturation at 95°C for 3 min, followed by 40 cycles of 95°C for 10 s, 52°C for 20 s, and 62°C for 30 s). The transcript expression of each target gene was normalized to expression levels of two housekeeping genes: threonine-tRNA ligase and glyceraldehyde-3-phosphate dehydrogenase, as reported earlier (17). The expression of each target gene in the *Pf*CDPK2 KO C1 parasite, relative to that in the WT, was calculated using Bio-Rad CFX Manager, and the data were analyzed using the R statistical analysis package (version 3.3.2) (47). NRT controls (without addition of reverse transcriptase) were prepared from the RNA samples to rule out any carryover contamination of DNA in the cDNA preparation and were amplified with either a ThrRS- or GAPDH-specific primer pair.

For stage-specific transcript expression of the *Pf*CDPK2 gene, the WT NF54 parasite culture was synchronized with 5% sorbitol followed by purification of late schizonts using the Percoll-sorbitol method. The purified schizonts were incubated with fresh RBCs for 4 h to allow invasion to take place. The newly invaded rings were synchronized with 5% sorbitol treatment to destroy mature parasites. The cultures were harvested at the ring (9 to 13 hpi), trophozoite (32 to 36 hpi), and schizont (44 to 48 hpi) stages. Template cDNA was prepared as described above.

### Western blotting for stage-specific CDPK2 expression.

The *Pf*CDPK2 HA-tagged and WT parasites were synchronized as described above for the stage-specific RT-qPCR experiment. The ring (15 to 21 hpi), trophozoite (27 to 33 hpi), and schizont (42 to 48 hpi) stages of the two parasite groups were harvested, and parasites were released from the RBCs by saponin treatment. The parasite pellets were washed three times with cold 1× phosphate-buffered saline (PBS) and lysed in the radioimmunoprecipitation assay (RIPA) buffer of the following composition: 150 mM NaCl, 1.0% NP-40, 0.5% sodium deoxycholate, 0.1% SDS, 50 mM Tris (pH 8.0), 1 mM phenylmethylsulfonyl fluoride, and 1× protease inhibitor cocktail (Roche Life Sciences, Indianapolis, IN) for 1 h on ice. The tubes were centrifuged at 14,000 × *g* for 20 min at 4°C. The lysate was loaded on an SDS-PAGE gel along with 1× NuPAGE-lithium dodecyl sulfate sample buffer (Thermo Fisher Scientific, Grand Island, NY). The separated proteins were transferred onto a polyvinylidene difluoride membrane followed by blocking with 5% skimmed milk in TBST (1× Tris-buffered saline [KD Medical, Columbia, MD] with 0.1% Tween 20) for 1 h at room temperature. The membrane was incubated with anti-HA antibody (Sigma-Aldrich, St. Louis, MO) at a 1:1,000 dilution or anti-CDPK1 antibody at a 1:2,000 dilution in blocking buffer for 1 h at room temperature followed by 3 washes with TBST. The membrane was then incubated with anti-rabbit secondary antibody conjugated with horseradish peroxidase (HRP; Sigma-Aldrich, St. Louis, MO) in the blocking buffer for 1 h at room temperature followed by 3 washes with TBST. The blot was incubated for 5 min with the SuperSignal West Dura extended duration substrate (Thermo Fisher Scientific, Grand Island, NY) and exposed on a HyBlot CL film followed by development with a Kodak X-Omat 2000A processor.

### Parasite growth rate experiment.

The *Pf*CDPK2 KO clones (C1 and C13) and the WT parasites were synchronized with 5% sorbitol treatment and seeded, 24 h postsynchronization, at 0.1% parasitemia, 2% hematocrit in a 96-well plate. The parasite samples were collected every 24 h for 7 days. The parasite growth rate was estimated using a fluorescence-based SYBR green assay as described earlier (48, 49).

### IFA.

*Plasmodium falciparum*-infected RBCs were smeared on a glass slide for the IFA experiments. The slides were fixed with 4% paraformaldehyde in 1× PBS for 20 min at room temperature. The slides were then blocked with 5% bovine serum albumin in 1× PBS containing 0.07% saponin for 45 min at room temperature. The slides were incubated with rabbit anti-*Pf*s230 antibodies (kindly provided by David Narum, LMIV, NIAID, NIH) or rat anti-α-tubulin II antibodies or mouse anti-band 3 antibodies (Santa Cruz Biotechnolology, Dallas, TX) in blocking buffer without saponin for 1 h at room temperature. The slides were washed three times with 1× PBS for 5 min each, followed by incubation with anti-rabbit Alexa Fluor 594, anti-rat Alexa Fluor 488, or anti-mouse Alexa Fluor 408 (Thermo Fisher Scientific, Grand Island, NY) in the blocking buffer without saponin for 50 min at room temperature. The slides were washed three times with 1× PBS for 5 min each. The slides were mounted with Vectashield HardSet Antifade mounting medium with or without 4′,6-diamidino-2-phenylindole (DAPI; Vector Laboratories, Burlingame, CA). The slides were incubated for 20 min at room temperature and then sealed with Cytoseal 60 (Electron Microscopy Sciences, Hatfield, PA). The slides were protected from light and stored at 4°C. The images were acquired with a Zeiss 880 confocal microscope with a 60×, 1.4-numerical aperture objective lens.

### α-Tubulin II peptide synthesis and generation of antisera.

The sequence encompassing the 10 C-terminal amino acids of α-tubulin II (CDGEGEDEGYE) (PlasmoDB no. PF3D7_0422300) supplemented with cysteine residue at the N terminus was synthesized by a solid-phase method using Fmoc chemistry (50) on an automated peptide synthesizer (model 433A; Applied Biosystems). After the trifluoroacetic acid cleavage step, the synthetic peptide was purified to homogeneity by reverse-phase (RP) high-performance liquid chromatography (HPLC) and lyophilized. To increase the immunogenicity of the peptide, it was conjugated to keyhole limpet hemocyanin (KLH; Thermo Fisher Scientific, Grand Island, NY) through the N-terminal cysteine residue by using the cross-linking reagent *m*-maleimidobenzoyl-*N*-hydroxysuccinimide ester (MBS; Thermo Fisher Scientific, Grand Island, NY). Antibodies against α-tubulin II were raised in rats following the guidelines of the Animal Safety Protocol (ASP) LMVR 1 approved by the Animal Care and Use Committee (ACUC), NIAID, NIH. Rats were immunized with the KLH-peptide conjugate under the following protocol: priming was done with 100 µg of the conjugate in Freund’s complete adjuvant (FCA; Sigma-Aldrich, St. Louis, MO), and the emulsion was injected subcutaneously. After 21 days, the rats were boosted with 50 µg of the conjugate in Freund’s incomplete adjuvant (FICA; Sigma-Aldrich, St. Louis, MO), and the emulsion was injected subcutaneously. A second booster was given 21 days after the first, following the same parameters. The test sera were collected from the rats 8 days after the second boost. The sera were tested in an IFA that stained male gametocytes and not female gametocytes.

### Gametocyte culture, gametogenesis, and mosquito infection.

The mixed-stage WT and the *Pf*CDPK2 KO parasites were set up at 2% parasitemia, 5% hematocrit, and gametocytogenesis was induced as described previously (18, 19). For the gametocytogenesis experiments, RPMI medium with human serum was used, as described earlier, without gentamicin. The medium was changed daily without adding fresh RBCs, and the culture was allowed to crash. The culture was monitored to ensure the presence of gametocytes. Gametogenesis was induced in day 14 to 16 gametocytes by resuspending the cultures in 50% human serum and dropping the temperature to room temperature. After 15 to 20 min of incubation, male gametocyte exflagellation was visualized under a bright-field microscope with a 40× objective.

For mosquito infections, day 14 to 16 gametocytes were fed to 4- to 6-day-old female *Anopheles stephensi* Nijmegen mosquitoes by standard membrane feeding. The development of the oocysts in the mosquito midgut was checked 7 to 9 days postinfection, as described elsewhere (51).

### Treatment and purification of gametocytes to detect *Pf*CDPK2 by Western blotting.

Gametocyte cultures were set up as described above. The asexual-stage parasites were killed by treatment with 50 mM NAG (22, 23) and 20 units/µl of heparin (24) on the fifth day for 4 consecutive days. The gametocytes were allowed to develop with daily medium changes. On the 14th day of the setup, the culture was centrifuged at 241 × *g* for 3 min to remove all the debris. The gametocyte-infected RBCs were purified by the Percoll-sorbitol method. The purified gametocytes were washed two times with RPMI 1640 medium with l-glutamine, 25 mM HEPES, and 50 µg/ml hypoxanthine (KD Medical, Columbia, MD). The gametocytes were released from the RBCs by treatment with 0.1% saponin in 1× PBS. The pellet was washed 3 times with 1× PBS and kept at −80°C until further processing. The samples for the Western blot assays were prepared as described above. The parasite lysate was probed with anti-HA antibody (Sigma-Aldrich, St. Louis, MO) at a 1:1,000 dilution, anti-CDPK1 antibody at a 1:2,000 dilution, or anti-AMA1 mouse monoclonal 2E3 antibody at a 1:1,000 dilution.

Additional information regarding our materials and methods is presented in Text S1 in the supplemental material.

10.1128/mBio.01656-17.1 TEXT S1  Supplemental materials and methods. Download TEXT S1, DOCX file, 0.02 MB. Copyright © 2017 Bansal et al. 2017 Bansal et al. This content is distributed under the terms of the Creative Commons Attribution 4.0 International license.
