# Supplementary material for: Plasmodium falciparum Calcium-Dependent Protein Kinase 2 Is Critical for Male Gametocyte Exflagellation but Not Essential for Asexual Proliferation
Source: mBio. 2017 Oct 17;8(5):e01656-17. doi: 10.1128/mBio.01656-17 (PMC5646254; doi:10.1128/mBio.01656-17)
Supplement: TABLE S1 [file mbo005173533st1.docx]

**Supplementary Table 1. Details of primers used in the study**

| **No.** | **Gene**  **(PlasmoDB number)** | **Oligo name** | **Oligo sequence (5'-3')** |
| --- | --- | --- | --- |
| 1 | CDPK2 (PF3D7_0610600) | F3 | CATGTGTAAATATATTGTTCTACTAAAGGAC |
| 2 | CDPK2(PF3D7_0610600) | R1 | CCCTACCTGAACATAATTCCAT |
| 3 | CDPK2(PF3D7_0610600) | R2 | CTATTGTAGCAGCCAGG |
| 4 | CDPK2(PF3D7_0610600) | R3 | ATGCGCCCTAGGTTTTTTTTTGCTCATCATAAGCATGAATTC |
| 5 | CDPK1(PF3D7_0217500) | CDPK1RTF | GGAAGAATTAGCAAATTTATTTGGTTTGACATC |
| 6 | CDPK1(PF3D7_0217500) | CDPK1RTR | ATGTTAACGAATTCATCAAAGTCAATCATGT |
| 7 | CDPK2(PF3D7_0610600) | CDPK2RTF | GGAACAGGAGAATTTACAACGAC |
| 8 | CDPK2(PF3D7_0610600) | CDPK2RTR | TGTATACATAATAACACCACTAGACCAG |
| 9 | CDPK3(PF3D7_0310100) | CDPK3RTF | CACGAAATATTGAGCATGGTAAAGAAGG |
| 10 | CDPK3(PF3D7_0310100) | CDPK3RTR | CAGCGTCCATTGTAAGACATCTTTTTATTAAATC |
| 11 | CDPK4(PF3D7_0717500) | CDPK4RTF | ATACTTCTCTCAGGGTGCCC |
| 12 | CDPK4(PF3D7_0717500) | CDPK4RTR | CTTATCACTAATTTTTTTGAATTGTGGTAAATCG |
| 13 | CDPK5(PF3D7_1337800) | CDPK5RTF | GGAGGTCGAAGATATGGATACGAATAG |
| 14 | CDPK5(PF3D7_1337800) | CDPK5RTR | TATCGGCTAACGTACTCTTTGTCG |
| 15 | CDPK6(PF3D7_1122800) | CDPK6RTF | CCTCCCGTAGATAAGAATATATTATCTATCG |
| 16 | CDPK6(PF3D7_1122800) | CDPK6RTR | ATCTGCTTCAATAAATCCCAATACATTTGC |
| 17 | CDPK7(PF3D7_1123100) | CDPK7RTF | AGTCCTAAAAAAGATATATAAAGAACTAGGTAGTAG |
| 18 | CDPK7(PF3D7_1123100) | CDPK7RTR | TTTAAAAATAATCTTTCTCCCCACAACCC |
| 19 | PKA(PF3D7_0934800) | PKARTF | AATCATCCATTTTGTGTAAATTTACATGG |
| 20 | PKA(PF3D7_0934800) | PKARTR | CTTTTGTTTCTTCTTAAAAATGTAAAAAATTCTCC |
| 21 | PKG(PF3D7_1436600) | PKGRTF | AAAGGGAATGAAAGAAATAAAAAGAAGGC |
| 22 | PKG(PF3D7_1436600) | PKGRTR | CATATCAATATCTTCTGAAAGCTTTTCCC |
| 23 | PKB(PF3D7_1246900) | PKBRTF | CACAATAGAAGAAATGATGTTCTTTTTTACG |
| 24 | PKB(PF3D7_1246900) | PKBRTR | GAGAGCGCAATTAGCCATATTG |
| 25 | PI3K(PF3D7_0515300) | PI3KRTF | CCCCTTCAATTTGTTTGTGAAACAG |
| **No.**  26 | **Gene**  **(PlasmoDB number)**  PI3K(PF3D7_0515300) | **Oligo Name**  PI3KRTR | **Oligo sequence (5'-3')**  ATCACATTTGTTATACTTATTATCATCACATTTGTT |
| 27 | GAPDH(PF3D7_1462800) | GAPDHRTF | GGAAGGAAAGATATCGAAGTAG |
| 28 | GAPDH(PF3D7_1462800) | GAPDHRTR | GGGTTACCTCACATGG |
| 29 | ThrRS(PF3D7_1126000) | TtRNALRTF | CTTGGGAACTGCAGAGTAGAATTT |
| 30 | ThrRS(PF3D7_1126000) | TtRNALRTR | TAAAAATCCTCCGAACAATTTTTCTAAACTAC |
| 31 | HSP90(PF3D7_0708400) | HSP90RTF | CGTACAAAGATCACCGAATTACTCC |
| 32 | HSP90(PF3D7_0708400) | HSP90RTR | GGTGATATAGTAAATATCCTTTTGGTTTTCC |
| 33 | CDPK2 (PF3D7_0610600) | F1 | GGACATATAATAACGTATCAGCCAAAG |
| 34 | CDPK2 (PF3D7_0610600) | R8 | AATTTTTTAATAATGTAGATGAAAGC |
| 35 | CDPK2 (PF3D7_0610600) | R8WT | GATTTTTTAATAAAGTGGAAGATAAT |
| 36 | CDPK2 (PF3D7_0610600) | R9 | CTGGAACATCATATGGATAAGC |
| 37 | CDPK2 (PF3D7_0610600) | Ck2seqHA1 | GCTCTTGACGTTGATAATAGTG |
| 38 | - | LNHA2 | ATTGGGGTGATGATAAAATGAAAG |
| 39 | GDV1 (PF3D7_0935400) | GDV1F2 | ACGAAATACTGAACATAGTATATTTCCTG |
| 40 | GDV1 (PF3D7_0935400) | GDV1R | GATGAGATACATATTATACACATATATTAGCATAC |
| 41 | - | pDC2A | AAAAACGTAATTTTTTCATTTGCCTTC |
| 42 | CDPK1(PF3D7_0217500) | Ck1F1 | ATTTTCTTTTCTGAACGTGTAACATG |
| 43 | CDPK1(PF3D7_0217500) | Ck1R1wt | TTCTAATAAAATATTTTCTGGTTTAATATCTCGA |
