# Supplementary figures and images for: Plasmodium falciparum Calcium-Dependent Protein Kinase 2 Is Critical for Male Gametocyte Exflagellation but Not Essential for Asexual Proliferation
Source: mBio. 2017 Oct 17;8(5):e01656-17. doi: 10.1128/mBio.01656-17 (PMC5646254; doi:10.1128/mBio.01656-17)

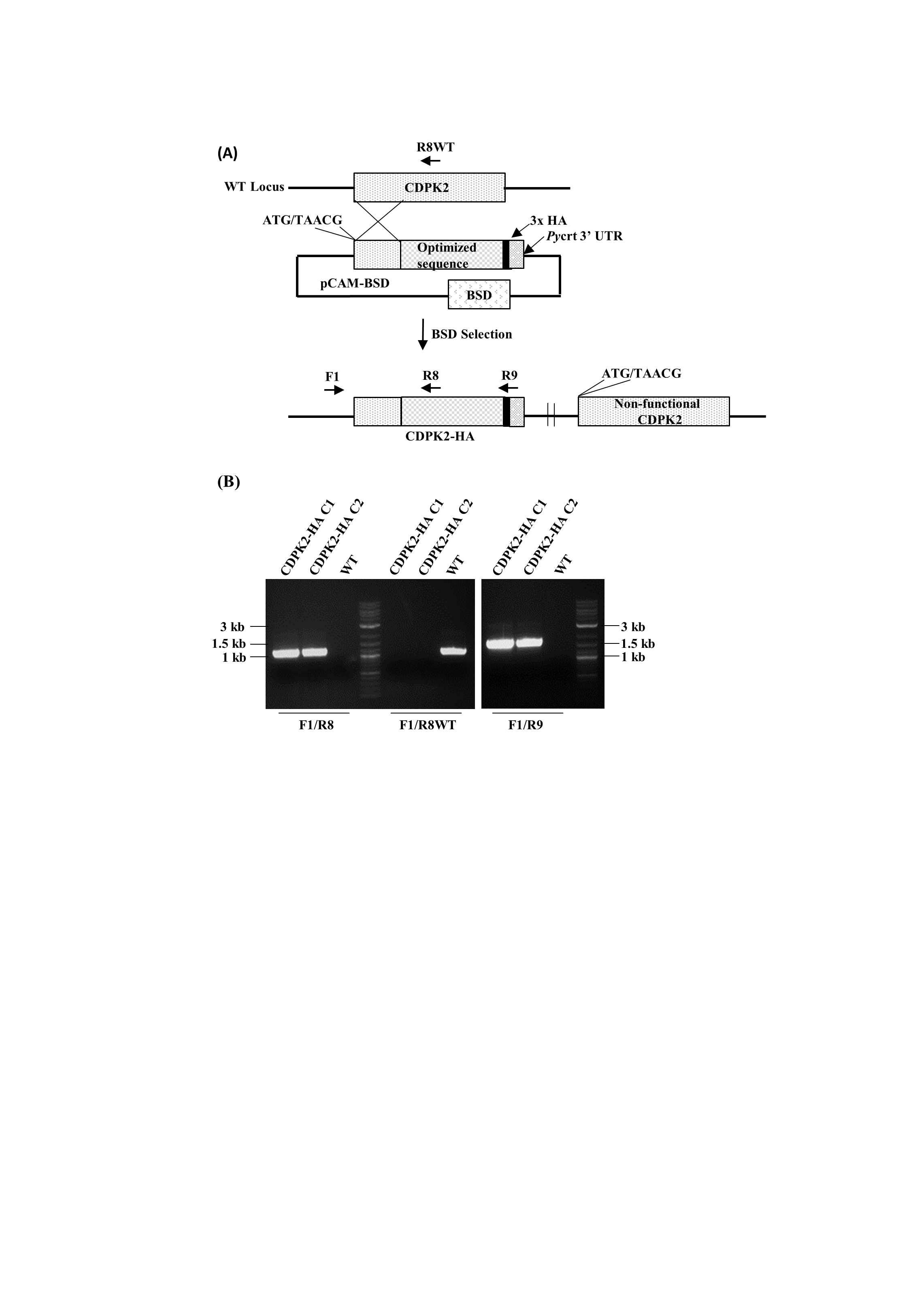

Supplement: FIG S1 [file mbo005173533sf1.tif]

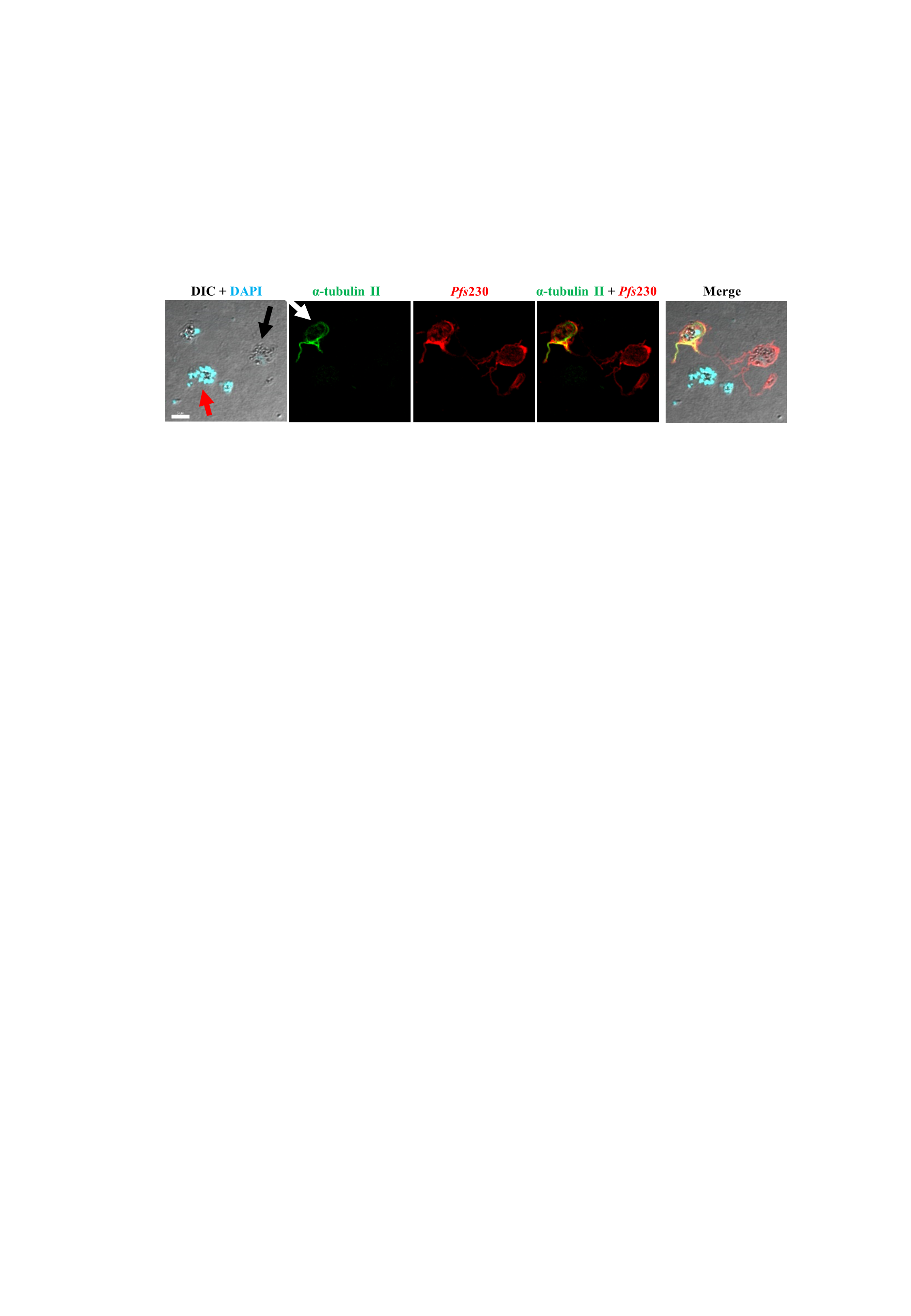

Supplement: FIG S2 [file mbo005173533sf2.tif]

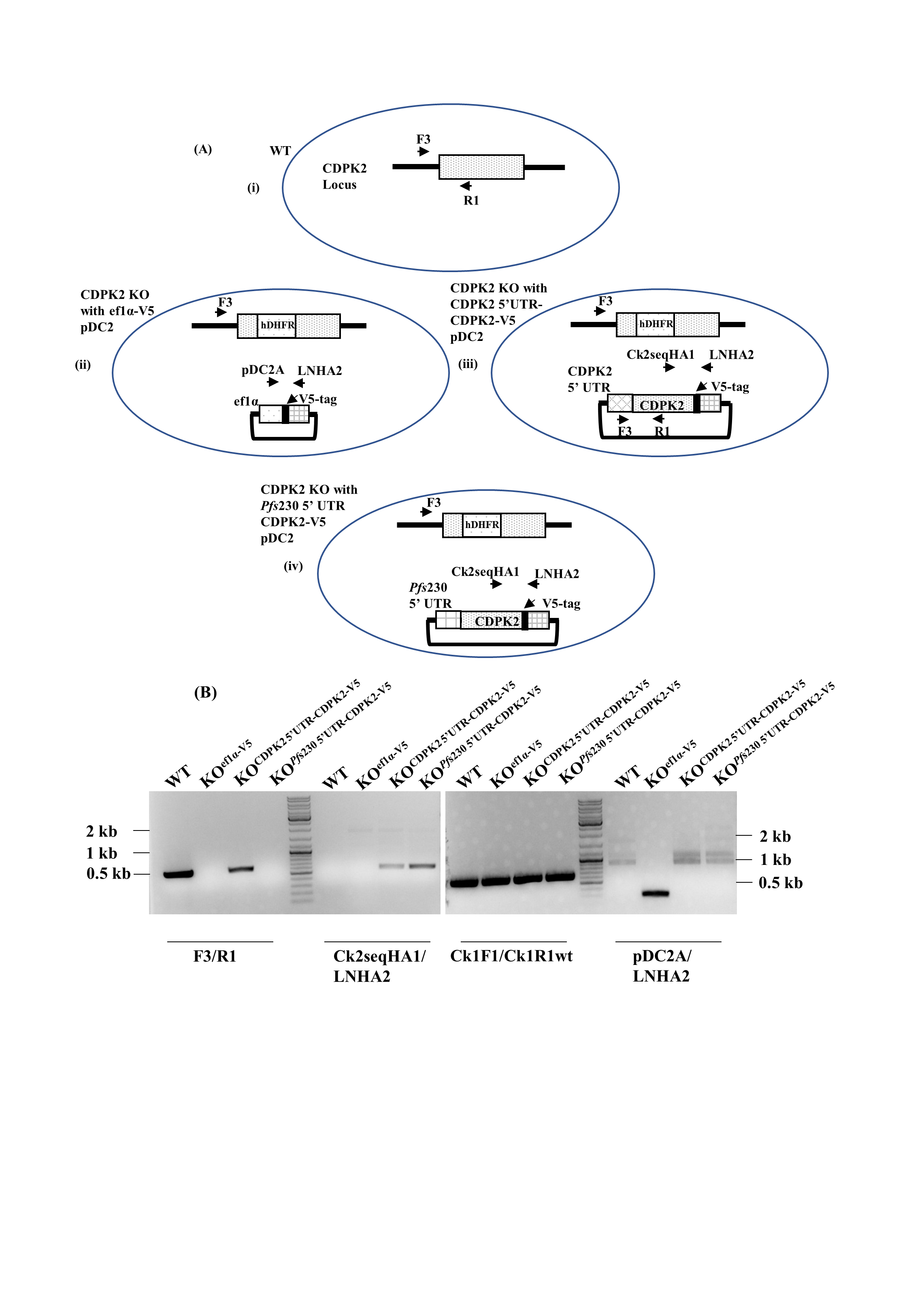

Supplement: FIG S3 [file mbo005173533sf3.tif]

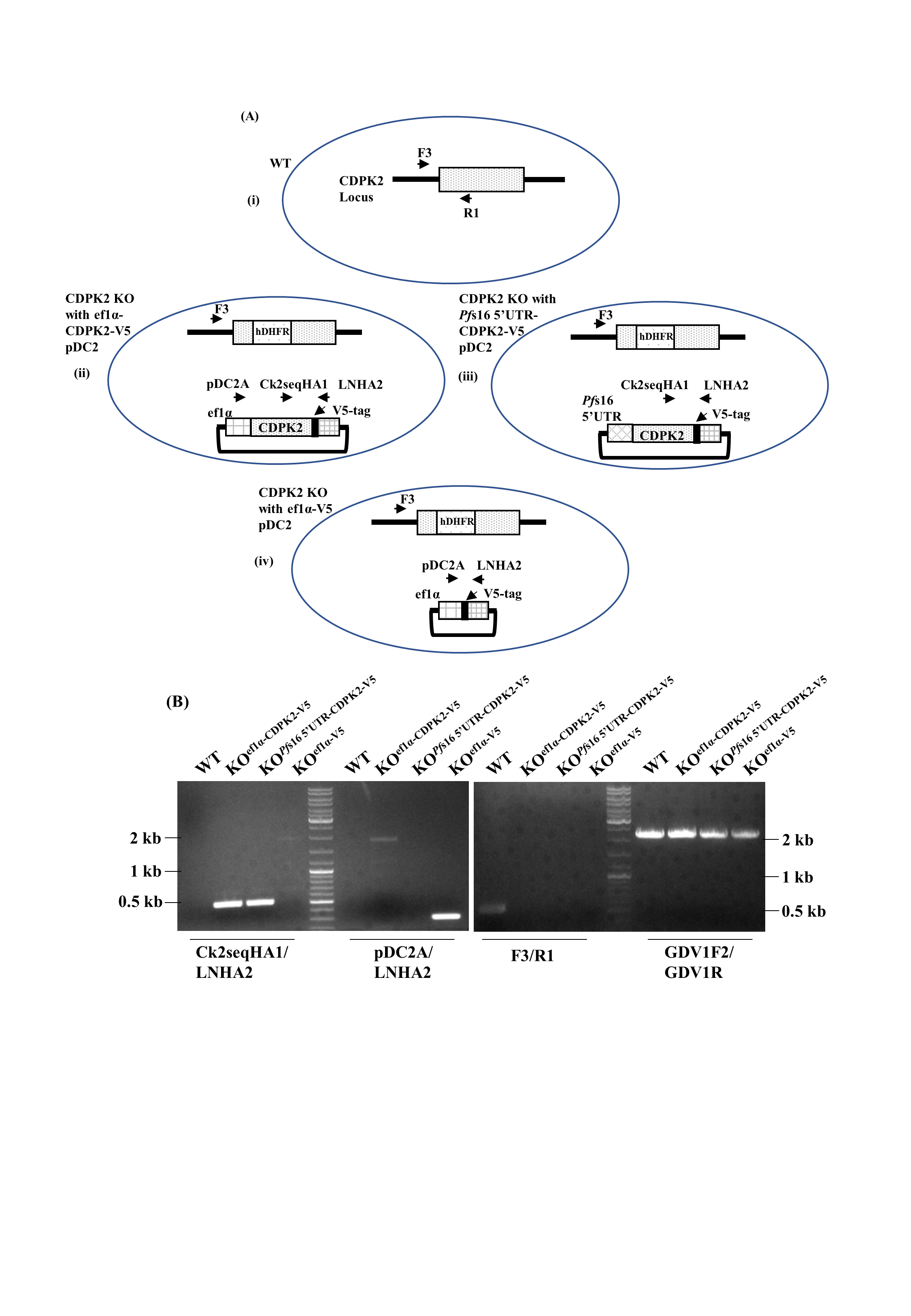

Supplement: FIG S4 [file mbo005173533sf4.tif]

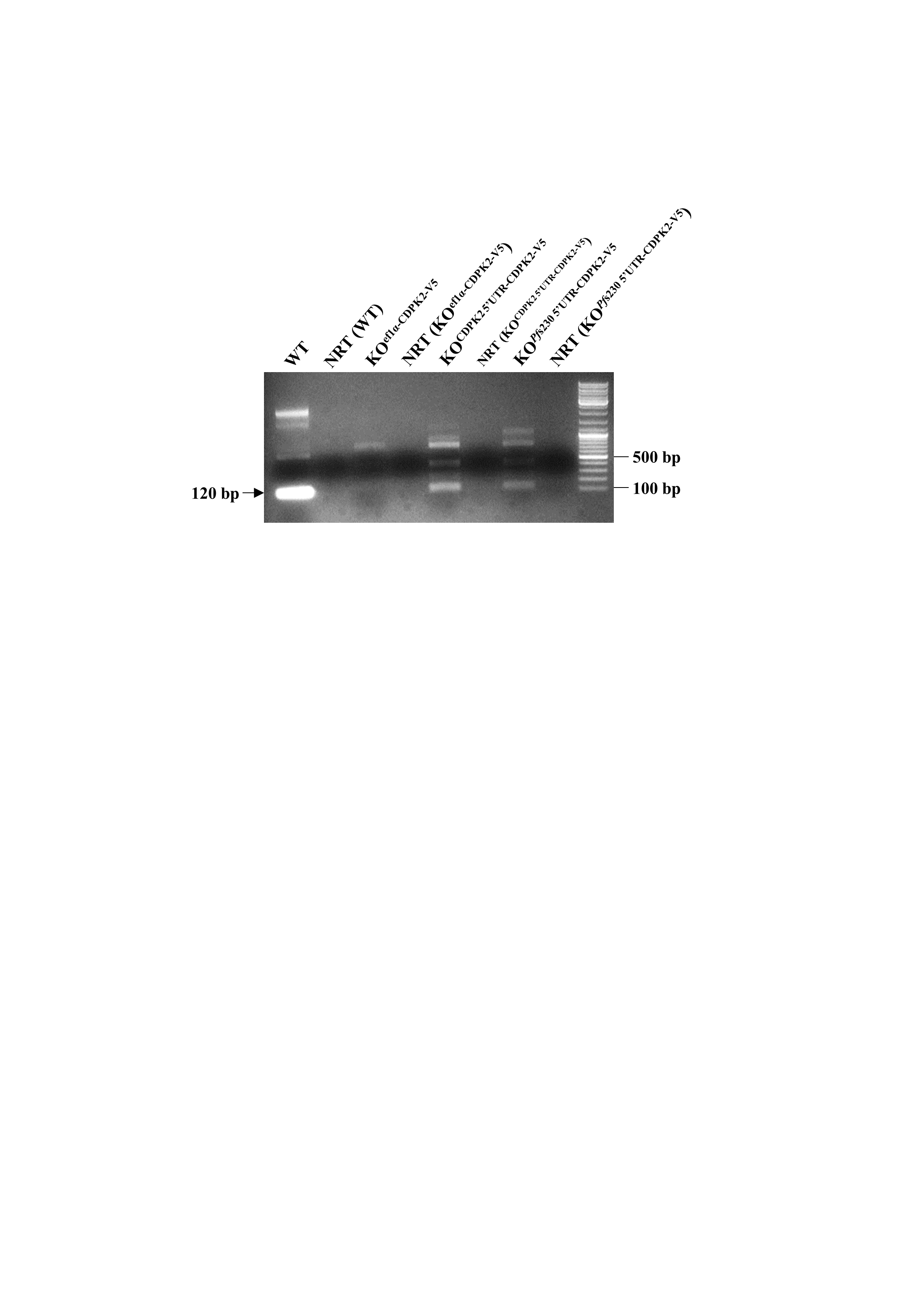

Supplement: FIG S5 [file mbo005173533sf5.tif]

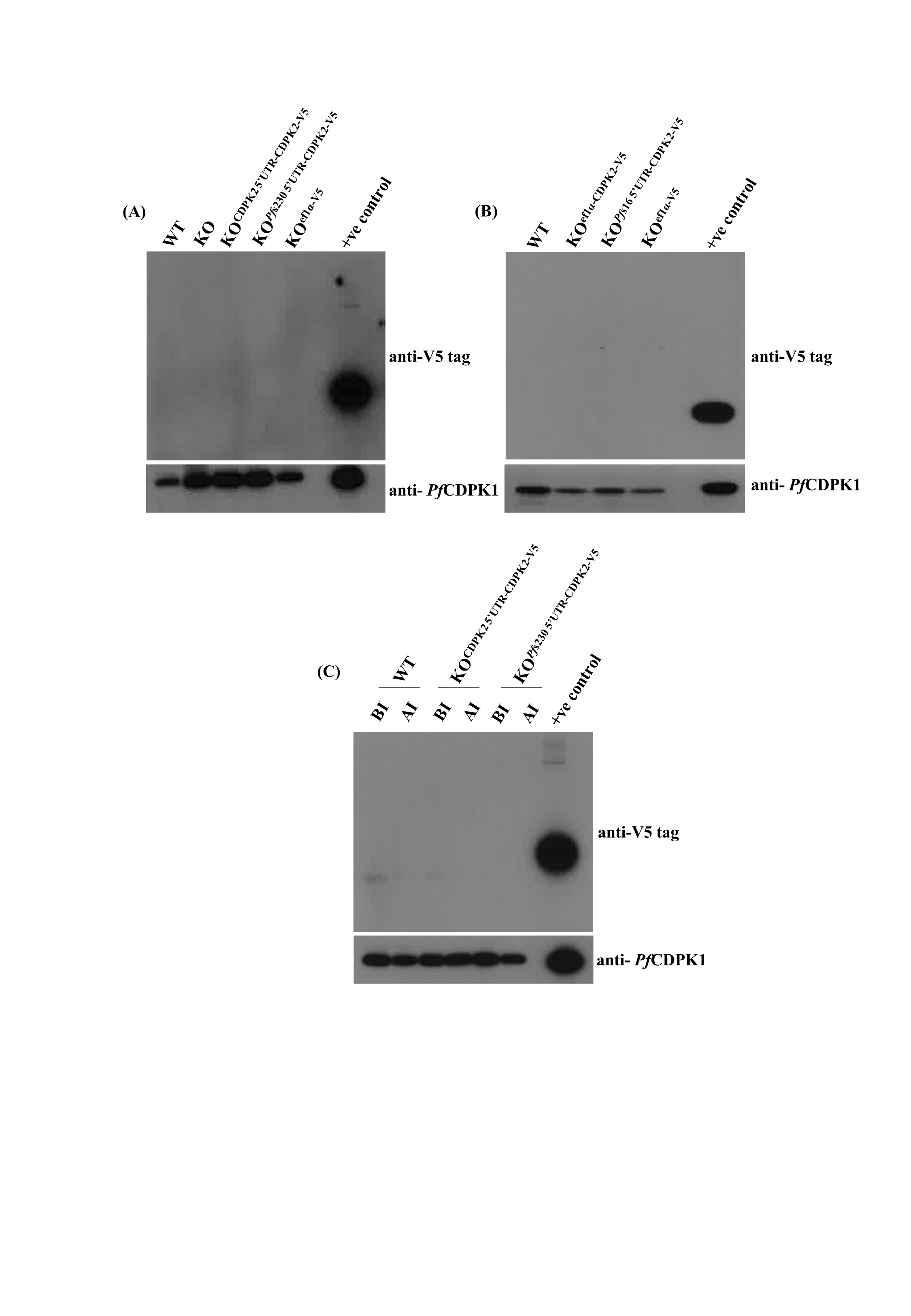

Supplement: FIG S6 [file mbo005173533sf6.tif]
