# Supplementary material for: Plasmodium falciparum Calcium-Dependent Protein Kinase 2 Is Critical for Male Gametocyte Exflagellation but Not Essential for Asexual Proliferation
Source: mBio. 2017 Oct 17;8(5):e01656-17. doi: 10.1128/mBio.01656-17 (PMC5646254; doi:10.1128/mBio.01656-17)
Supplement: TEXT S1 [file mbo005173533s1.docx]

**Supplementary Information**

**Materials and Methods**

**Construction of the CDPK2-HA tag plasmid.** A synthetic gene sequence for *Pf*CDPK2 (PlasmoDB no. PF3D7_0610600**)** was synthesized with the open reading frame disrupted (ATG replaced with TAACG) while keeping the following 423 nucleotides the same as the wild type sequence to allow for single recombination. The rest of the gene sequence was optimized (with the same amino acid sequence as the wild type) so as not to allow any recombination from the region. A 3x hemagglutinin (3x HA) tag (amino acid sequence: YPYDVPDYA) was attached at the 3’ end of the gene to follow the protein expression in the parasite lifecycle followed by a stop codon (TAA). The complete synthetic sequence was flanked at the 5’ and 3’ end by SacI and BamHI restriction sites, respectively. A 3’ UTR region of *P*. *yoelii* chloroquine transporter (*Py*crt 3’ UTR) (1) was amplified using the following oligo pair *Py*crt3’UTRF (5’ ATGCGCGGATCCTAGATACAACATATTTATTTTTTAAATATTTTTTTTAAATGCC 3’) and *Py*crt3’UTR (5’ AATCTGCAGGCGAAAGATATTTCAAAAATCTTAGCATAAG 3’). The underlined sequences are the restriction sites for BamHI and PstI, respectively. The amplified *Py*crt 3’ UTR was cloned at the 3’ end of the *Pf*CDPK2 synthetic gene sequence using the restriction enzymes BamHI and PstI. The resulting construct was cloned in the pCAM-BSD plasmid (provided by David Fidock) using the SacI and PstI restriction sites.

**Construction of plasmids for the *Pf*CDPK2 KO complementation.** The complete open reading frame of *Pf*CDPK2 was amplified using the following oligo pair pDC2cdpk2fwd (5’ ATGCGCCCTAGGATGGGAAATCACTTATCAGTAAATAAATTAAAAAGGAAAAAAAAAAAAAAAAG 3’) and pDC2cdpk2rev (5’ ATGCGCACGCGTTTTTTTTTTGCTCATCATAAGCATGAATTCATGGAAATC 3’). The underlined sequences are the restriction sites for AvrII and MluI, respectively. The amplified product was cloned in the pDC2 plasmid (generously provided by Richard Eastman) using AvrII and MluI in-frame with a V5 tag (G K P I P N P L L G L D S T) followed by hsp86 3’UTR. The expression of the episomal *Pf*CDPK2 was driven by either 1.5 kb of CDPK2 5’ UTR immediately upstream of the start codon, *Pfs*230 5’ UTR (2), ef1α promoter (3) or *Pf*s16 5’ UTR (4). The CDPK2 5’ UTR, *Pfs*230 5’ UTR and *Pf*s16 5’UTR were amplified using the following oligo pairs pDC2CDPK2PROfwd ATGCGCGGGCCCTAATATTTTAAAAATAAATATCACAAAAATAATATAAAATAATATAAAGTAAAATAAAATAAAAATATATTAAC *and* pDC2CDPK2PROrev *(*ATGCGCCCTAGGAATAAGTATTATTCTTTATATATACATATATATATATATATATATATATATATATATTATTTTGCACTATTTTTAATATATATTTTG); *Pfs*230UTRFWD *(*ATGCGCGGGCCCGAGCATATATACCCATGGTCCTTAAC *) and Pfs*230UTRREV: ATGCGCCCTAGGCGATCAAGAAGGATAAAAGAATGGTAGAAG *); Pf*s16UTRFWD (5’ATGCGCGGGCCCCAATATGAATTTATAGAGCAAATTTATATAGTTAAAAAAAAAAAA 3’) and *Pf*s16UTRREV (5’ ATGCGCCCTAGGTGTTGAAGAAAGTATAAATAGAAAAATGGCAAAA 3’), respectively. The underlined sequences are the restriction sites for ApaI (GGGCCC) and AvrII (CCTAGG). The empty parent plasmid without the *Pf*CDPK2 gene was used as a control for the complementation studies.

**REFERENCES**

1. Valderramos SG, Valderramos JC, Musset L, Purcell LA, Mercereau-Puijalon O, Legrand E, Fidock DA. 2010. Identification of a mutant PfCRT-mediated chloroquine tolerance phenotype in Plasmodium falciparum. PLoS Pathog 6:e1000887.

2. Eksi S, Suri A, Williamson KC. 2008. Sex- and stage-specific reporter gene expression in Plasmodium falciparum. Mol Biochem Parasitol 160:148-51.

3. Fernandez-Becerra C, de Azevedo MF, Yamamoto MM, del Portillo HA. 2003. Plasmodium falciparum: new vector with bi-directional promoter activity to stably express transgenes. Exp Parasitol 103:88-91.

4. Eksi S, Williamson KC. 2011. Protein targeting to the parasitophorous vacuole membrane of Plasmodium falciparum. Eukaryot Cell 10:744-52.
